# Promoter evolution of mammalian gene duplicates

**DOI:** 10.1186/s12915-023-01590-6

**Published:** 2023-04-13

**Authors:** Evgeny Fraimovitch, Tzachi Hagai

**Affiliations:** Shmunis School of Biomedicine and Cancer Research, George S Wise Faculty of Life Sciences, Tel Aviv University, 69978 Tel Aviv, Israel

**Keywords:** Promoter evolution, *Cis*-regulation, Gene duplication, Metazoan promoters, CpG Islands

## Abstract

**Background:**

Gene duplication is thought to be a central process in evolution to gain new functions. The factors that dictate gene retention following duplication as well paralog gene divergence in sequence, expression and function have been extensively studied. However, relatively little is known about the evolution of promoter regions of gene duplicates and how they influence gene duplicate divergence. Here, we focus on promoters of paralog genes, comparing their similarity in sequence, in the sets of transcription factors (TFs) that bind them, and in their overall promoter architecture.

**Results:**

We observe that promoters of recent duplications display higher sequence similarity between them and that sequence similarity rapidly declines between promoters of more ancient paralogs. In contrast, similarity in *cis*-regulation, as measured by the set of TFs that bind promoters of both paralogs, does not simply decrease with time from duplication and is instead related to promoter architecture—paralogs with CpG Islands (CGIs) in their promoters share a greater fraction of TFs, while CGI-less paralogs are more divergent in their TF binding set.

Focusing on recent duplication events and partitioning them by their duplication mechanism enables us to uncover promoter properties associated with gene retention, as well as to characterize the evolution of promoters of newly born genes: In recent retrotransposition-mediated duplications, we observe asymmetry in *cis*-regulation of paralog pairs: Retrocopy genes are lowly expressed and their promoters are bound by fewer TFs and are depleted of CGIs, in comparison with the original gene copy. Furthermore, looking at recent segmental duplication regions in primates enable us to compare successful retentions versus loss of duplicates, showing that duplicate retention is associated with fewer TFs and with CGI-less promoter architecture.

**Conclusions:**

In this work, we profiled promoters of gene duplicates and their inter-paralog divergence. We also studied how their characteristics are associated with duplication time and duplication mechanism, as well as with the fate of these duplicates. These results underline the importance of *cis*-regulatory mechanisms in shaping the evolution of new genes and their fate following duplication.

**Supplementary Information:**

The online version contains supplementary material available at 10.1186/s12915-023-01590-6.

## Background

Gene duplication introduces new gene copies and, as such, plays a central role in genome evolution and organismal complexity [1, 2]. For example, gene duplication has led to the expansion of various transcription factor families, including the homeobox gene family that plays central roles in embryonic development [3]. Gene duplication is also thought to be important for expanding the repertoire of restriction factors against rapidly evolving pathogens [4, 5], as observed in the recurrent duplication and diversification of the antiviral enzyme cytosine deaminase APOBEC3 in different mammalian clades, including bats and primates [6–9].

Many of the duplicated genes, however, are not fixed following duplication and do not evolve into a functional paralog [10, 11]. Successful gene retention is influenced by various factors, including dosage balance constraints [12], gene evolvability and inter-paralog interactions [13, 14], and gene expression level and gene length [15]. In addition, the mechanism of gene duplication, whether duplicates are products of whole-genome duplication or small-scale duplication events, can also impact the set of retained genes [16–18].

A successful gene retention often involves gene sub- or neo-functionalization and entails the incorporation of this gene into the cellular network. This includes complex processes from transcriptional regulation of the new gene to the interactions its protein product forms within the protein interaction network [14, 19, 20]. Previous works have used large-scale analyses or focused on specific gene subsets to study differences between gene duplicates at the level of coding sequence evolution, transcriptional divergence, and functional diversification [21–26]. However, despite their importance to gene function and evolution, relatively little is known on promoters of duplicated genes, how their characteristics shape gene duplication, retention and evolvability, and how their sequences and regulatory functions evolve following duplication.

As crucial elements for gene regulation, evolutionary changes in promoters can greatly impact gene function [27]. An analysis of promoter sequence evolution in primates found enrichment of positive selection in promoters of genes associated with particular pathways, such as neuronal development, pointing to the contribution of *cis*-regulatory changes to human evolution [28]. Another study suggested that rapidly evolving non-coding regions are enriched in the vicinity of recently duplicated genes [29], implying that accelerated promoter evolution may be related to gene duplicate sub- or neo-functionalization.

Mammalian promoters vary in their overall architecture (the regulatory elements embedded within them), the transcription factors (TFs) that bind them, and the number and positioning of transcription starting sites (TSSs) of their regulated genes [30]. Several promoter types have been proposed, based on the presence of certain regulatory elements, types of histone modifications, and mode of transcription of the genes under their control [30, 31]. Here, when characterizing promoter architecture in gene duplicates, we partition genes based on presence of CpG Islands. More than half of the promoters of coding genes in mammals are associated with regions of non-methylated DNA, called CpG islands (CGIs), where CpG dinucleotide frequency is higher in comparison with other regions along the genome [32]. These CGIs are thought to enable a transcriptionally permissive chromatin environment [33, 34] that opposes the repressive effects of methylation [35]. CGI-rich promoters constitute a major class of promoters that have a characteristic chromatin organization and that is linked with specific patterns of transcription initiation and gene expression [30, 36].

In this work, we analyze promoters of gene duplicates to investigate evolutionary changes between paralogs. For this, we compare sequence similarity, TF binding, and overall architecture between promoters of paralog genes. We partition paralogs by estimated duplication time and by inferred duplication mechanism to reveal trends of evolutionary conservation and divergence of their promoters. We next focus on recent duplications to characterize promoters and *cis*-*regulation* of new genes as well as to study associations between promoter characteristics and gene retention following duplication. Finally, we analyze the relationship between promoter architecture and the conservation of *cis*-regulation between gene duplicates.

## Results

### Following duplication, sequence similarity is rapidly lost between promoters of paralogs

To study the evolutionary patterns of gene duplication with respect to gene promoters, we focused on all pairs of paralogs in human and, separately, in mouse. For each paralog pair, we inferred the evolutionary time of duplication using either (1) phylogenetically based dating with gene tree topology from ENSEMBL [37], or (2) the rate of synonymous substitutions, dS, between the two paralogs (in their coding sequences) under the assumption it represents a molecular clock (see “Methods”). We also used a subset of these paralog pairs, where for each gene family composed of N genes, we chose a set of N-1 pairs. We quantified promoter sequence similarity between each pair of paralog genes, by employing a pairwise similarity score based on a local alignment using Kimura’s 2-parameter model (K2P) [38] on a region upstream of the transcription start site (TSS) (See “Methods”).

**Fig. 1:**
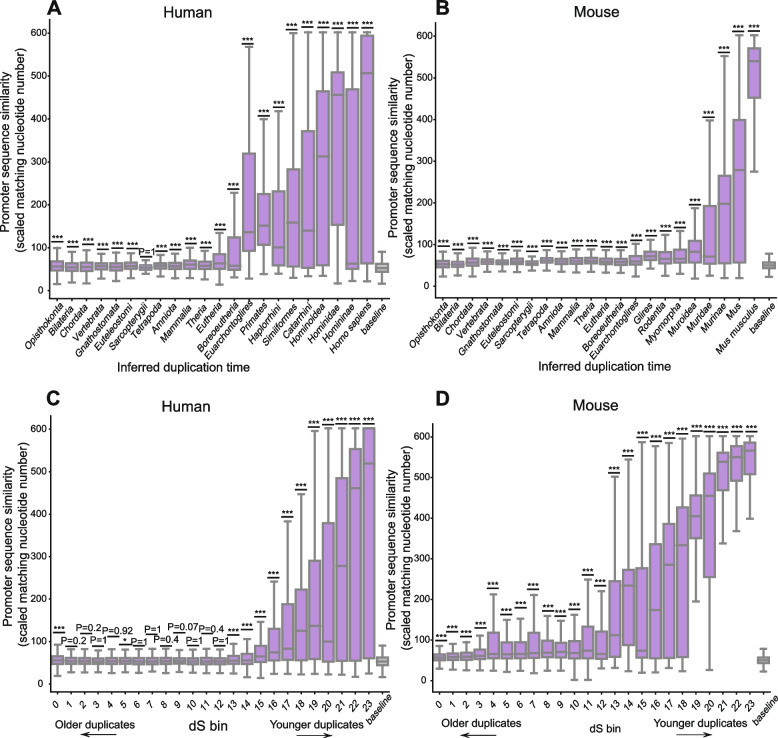
Sequence similarity between promoters of paralogs in human and mouse genomes. **A** Sequence similarity scores between promoter regions of human paralogs, where paralogs are partitioned based on their inferred duplication time (at each TMRCA (time to most recent common ancestor) from Opisthokonta to Homo Sapiens). In each group of paralogs belonging to the same TMRCA, the distribution of similarity scores is compared to that of randomly matched set of human gene promoters. Comparison between the distributions was preformed using a Mann–Whitney one-sided test and corrected by FDR. **B** As in **A**, only with mouse paralogs. **C** As in **A**, but with partitioning of paralogs based on their dS values (synonymous substitution rate between paralogs). Paralogs are binned into equal-sized bins. Left-most bins represent the highest dS values, and likely the oldest duplicates, while right-most bins represent lowest dS values. **D** as in **C**, only with mouse paralogs. (***P < 0.001, **P < 0.01, *P < 0.05). Group size numbers appear in Additional File 2: Table 1

A comparison of promoter sequences between paralog pairs shows that sequence similarity between paralog promoters is usually low (Fig. 1A–D). When dividing paralogs based on their inferred duplication time, we observe that a fraction of paralogs originating in recent duplication events has higher promoter sequence similarity. This relatively higher similarity between promoters of recent paralogs drops in evolutionarily older duplicates, suggesting that gene promoter regions diverge rapidly in sequence following duplication. We note that while sequence similarity between paralog pairs is low, it is still slightly, but significantly, higher in many cases than the “baseline similarity” observed between promoter sequences of unrelated pairs of genes (as determined using FDR-corrected Mann–Whitney test, see Fig. 1 for detailed *P*-values for each group of paralogs). This higher than random similarity may suggest a residual conservation between paralog promoter sequences. We repeated this analysis with promoter sequences of various lengths—100, 300, 500, and 1000 bp upstream of the TSS, observing similar trends (We show as examples, the analyses shown in Fig. 1B,D with additional promoter lengths in Additional File 1: Fig. S1-2). We also repeated this analysis with a reduced number of paralogs, such that for each gene family only paralogs inferred to be most closely related will be compared in their promoter sequence similarities, that resulted in similar trends (Additional File 1: Fig. S3-4).

We next looked at ancient paralogs that have unusually conserved promoter sequences (with sequence similarity score above 200, out of the maximum possible of 300). When testing for enriched functions using g:Profiler [39], several pathways involving the genes of these ultra-conserved promoters emerge (See full list of genes and the enrichment analysis in Additional File 2: Table 3A-D). Interestingly, among these pathways, the strongest signals observed in both human and mouse paralogs are histones (H3 and 4), genes associated with keratin and several complement-related genes. Other gene groups that appear in both species but are strongly enriched in only one of them include olfactory receptors, zinc finger proteins, GPCR associated proteins, and several types of enzymes.

### Promoter sequence similarity between paralogs is lower than between orthologs

We next quantified promoter sequence similarity between orthologs in the same manner as between paralogs, and compared this with sequence similarity between paralog promoters. We performed this analysis on promoter sequences of paralogs and orthologs that have had a similar time of divergence between the compared genes: In the case of orthologs, we used one-to-one orthologs between human and mouse, whereas for paralogs we used gene pairs that are predicted to have duplicated in the last common ancestor of primates and rodents. We observe that orthologs are more conserved in promoter sequence than paralogs (Additional File 1: Fig. S5). This is observed both for the group of paralogs found in the human genome, as well as for the group of paralogs in mouse (*P*-value = 6.4 × 10^−173^ and 3.7 × 10^−30^, respectively, FDR-corrected Mann–Whitney test). This higher conservation in orthologs is expected given the different evolutionary forces acting on ortholog and paralog genes (and on their *cis*-regulatory regions), and is in agreement with a previous analysis on divergence in gene expression of orthologs versus paralogs [22].

### Promoter sequence similarity of retrotransposition-mediated duplications versus segmental duplications

The conservation of promoter sequences between paralogs is dependent, among other factors, on duplication of the promoter region itself. In some cases, such as in retrotransposition-mediated duplications, the promoter is not part of the duplicated segment, while in segmental duplications the promoter region can be fully, or partially, duplicated. Thus, different duplication mechanisms may lead to different levels of promoter conservation. To test this, we divided the duplicated genes into those inferred to be a product of retrotransposition and those that are a result of other duplication mechanisms (i.e., segmental duplications). This was done by comparing gene structure and exon numbers between the two duplicated genes, following a previous study [23] (see “Methods”). We note that this method of distinguishing between duplication mechanisms is more accurate in relatively recent duplication events, since the gene structure may change during longer evolutionary periods, limiting the ability to accurately infer retrotransposition events. We also note that not all paralog pairs pass these criteria (of either being identified as segmental or retrotransposition-mediated duplications), and are thus removed from the following analyses.

**Fig. 2:**
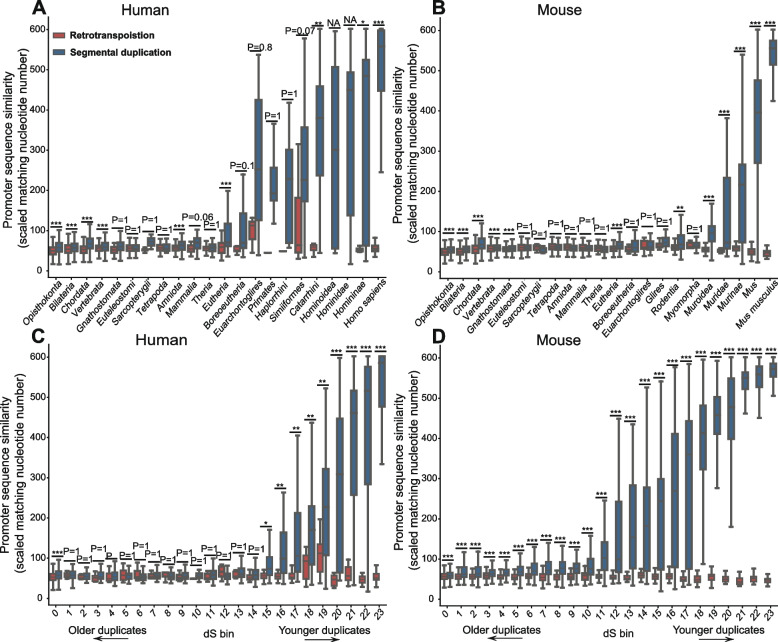
Sequence similarity between promoters of paralogs in segmental *versus* retrotransposition-mediated duplications. **A** Sequence similarity score between promoter regions of human paralogs, where paralogs are partitioned based on their inferred duplication time (as in Fig. 1) and based on inferred duplication mechanism: retrotransposition or segmental duplication, in red and blue, respectively. In each group of paralogs belonging to the same TMRCA, the distribution of similarity scores is compared between retrotransposition-mediated and segmental duplications. Comparison between the distributions was preformed using a Mann–Whitney one-sided test and corrected by FDR. (NA—only segmental duplications exist in this group). **B** As in **A**, but with mouse paralogs. **C** As in **A**, but with partitioning of paralogs based on their dS values (synonymous substitution rate between paralogs). Paralogs are binned into equal-sized bins. Left-most bins represent the highest dS values, and likely the oldest duplicates, while right-most bins represent lowest dS values. **D** as in **C**, only with mouse paralogs. (***P < 0.001, **P < 0.01, *P < 0.05). Group size numbers appear in Additional File 2: Table 2

When comparing promoter sequences of paralog pairs that are products of retrotransposition versus those originating from segmental duplication, we indeed observe trends that clearly distinguish between the two groups (Fig. 2A–D): Paralogs originating from retrotransposition-mediated duplications show significantly lower sequence similarity in recent duplication times in comparison with paralogs from segmental duplications. The differences in sequence similarity between promoters of paralogs originating in segmental duplications and retrotransposition-mediated duplications largely vanish in more ancient duplications, where both duplication classes display low similarity between paralogs. Thus, after sufficient time from duplication, promoters of most duplicates accumulate mutations in a manner that renders their sequences dissimilar, regardless of the initial duplication mechanism. We note that these trends are observed also when we change the promoter length and when we reduce the number of paralogs to a set of the most closely related pairs (See Additional File 1: Fig. S6-7 for analyses where we reduce the numbers of paralogs and use promoter length of 300 and 1000 bp in human and mouse, respectively). As expected, the differences between the two groups (retrotransposition-mediated and segmental duplications) are reduced in longer promoters, likely since the promoter region with similarity between the paralog is adjacent to the TSS.

Finally, we also compared the tendency of duplicates to reside on the same chromosome. We observe a trend where younger duplicates and duplicates originating in segmental duplications to have a higher tendency to reside on the same chromosome in comparison with older duplications and retrotransposition-mediated duplications (Additional File 1: Fig. S8).

### Retrotranspositioned gene copies are lowly expressed and have few TFs that bind their promoters

Next, we focused on recent retrotransposition events in human and mouse genomes (those that duplicated from the split between rodents and primates), where we can infer which of the paired paralogs is likely to be the original and which is likely to be the retrotransposed copy (see “Methods”). Utilizing this information, we compared promoter characteristics and *cis*-regulation between the original and the retrocopy genes.

**Fig. 3:**
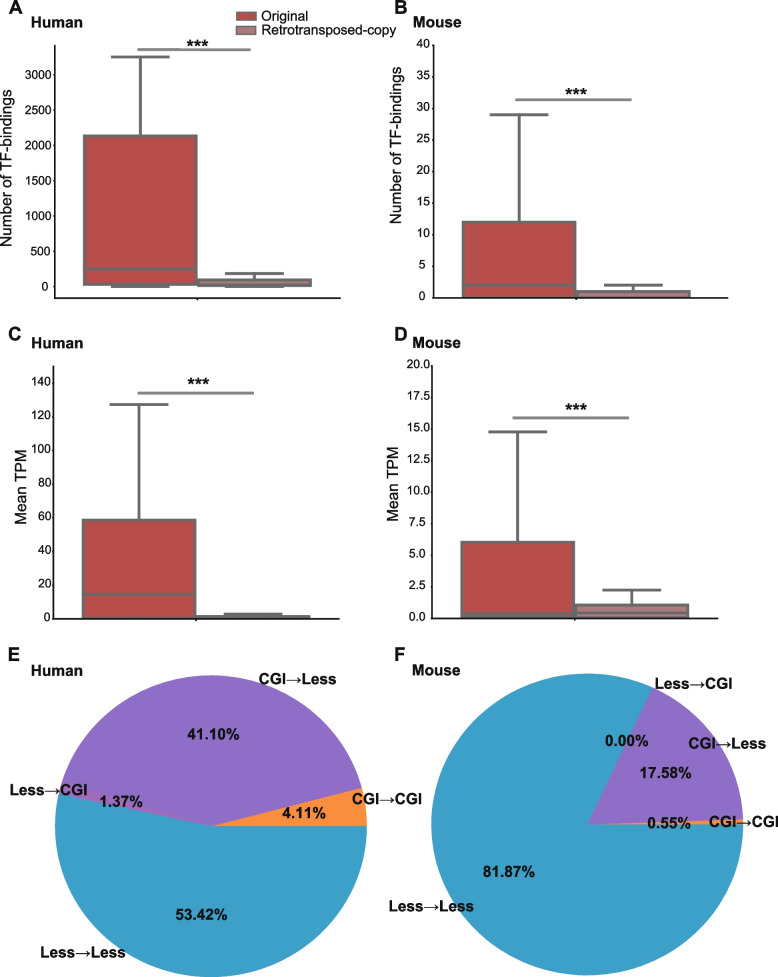
Promoter characteristics and gene expression in retrotransposition-mediated duplication. **A**,**B** The number of TF binding events in promoters of human and mouse genes, in gene duplicates that recently duplicated through retrotransposition (151 and 196 pairs, respectively). The left distribution corresponds to the original gene, and the right to the retrocopied gene. Comparison between the distributions was preformed using a Mann–Whitney one-sided test. **C**,**D** Mean expression level of human and mouse genes, in gene duplicates that recently duplicated through retrotransposition. The left distribution corresponds to the original gene, and the right to the retrocopied gene. Comparison between the distributions was preformed using a Mann–Whitney one-sided test. **E**,**F** Pie charts showing the partition of gene duplicates that recently duplicated through retrotransposition in human and mouse, based on the promoter architecture of the original and the retrocopied genes. Each gene in these pairs can either be a CGI gene or a CGI-less gene, yielding four possible combinations. The combination is denoted using an arrow pointing from the original gene to the retrocopy gene (for example, CGI->Less denotes the fraction of gene pairs that have CGI in promoters of the original gene and are depleted of CGI in promoters of the retrocopied gene). (***P < 0.001)

We first compared transcription factor (TF) binding in promoter regions of the original and the retrocopied paralogs. For this, we used Cistrome—a large dataset of ChIP-seq data that includes numerous TF-ChIP studies in both human and mouse in a diverse set of cells and tissues [39]. Following exclusion of several general TFs and insulators, we overlapped each gene’s promoter region with peaks of various TF-ChIP-seq data, yielding the set of TFs that are experimentally known to bind the proximal promoter region of each human and mouse gene (see “Methods” for details). With this data, we compared the total number of TFs that bind to the promoters of the original and the retrocopy genes across cells and tissues. We observed that a larger total number of TF binding events is recorded for the original genes and significantly fewer bindings are found for retrocopied genes in both human and mouse retrotranspositions (*P*-value = 9.03 × 10^−8^ and 2.67 × 10^−108^, respectively, Mann–Whitney test, Fig. 3A,B).

Next, we compared the expression of the original and the retrocopy genes, by studying the differences between paralogs in their expression across a large set of tissues in human and in mouse. For this, we used the GTEx data, which includes transcriptomics data from numerous tissues from a large number of human donors [40] to compare paralog expression in human tissues. Similarly, we used mouse transcriptomics BodyMap dataset [41] to compare paralog expression in mouse tissues. In both human and mouse, we observe that the original gene is more highly expressed across tissues than the retrocopy (*P*-value = 3.76 × 10^−10^ and 1.41 × 10^−8^, respectively, Mann–Whitney test, Fig. 3C,D).

The above observations suggest that recently retrotransposed genes are lowly expressed and that their expression is controlled by fewer TFs in comparison with the original gene.

### Retrotranspositioned gene copies differ from the originally copied gene in promoter architecture

We next focused on the type of promoters associated with the original and the retrocopied genes. Among both paralog genes, we identified which genes harbor CpG Islands (CGIs) in their promoters (with a significant overlap between CGIs and promoter regions—see “Methods” for details). We term these genes—“CGI genes.” All other genes were defined as “CGI-less genes.”

Across the genome, the majority of genes are CGI genes—56.9% in human and 57.2% in mouse. However, we observe a depletion of CGI genes in the original copy—45 and 18% in human and mouse (*P*-value = 0.14 and < 10^−20^, respectively, Fisher’s exact test). An even more prominent depletion of CGI is observed in promoters of the retrocopied genes—only 5.5 and 0.5% of the human and mouse retrocopies, respectively, harbor CGIs in their promoters (*P*-value < 10^−20^ in both human and mouse, Fisher’s exact test) (Fig. 3E,F). When looking at the partition of original and retrocopy pairs, in terms of their promoters, we observe that the largest group is composed of pairs where both original and retrocopied genes are CGI-less (53.4 and 81.9% of all recent retrotransposition events, in human and mouse, respectively). The next largest group includes genes where the original copy is a CGI gene, while the retrocopy is CGI-less (41.1 and 17.6%, in human and mouse, respectively).

Thus, the retrotransposed copy nearly always has a promoter that lacks CGI elements, regardless of whether the original gene has a CGI promoter or not. This is in line with the lower expression of the retrocopy gene in comparison with the original gene, which we reported above. We note that similar results are observed when looking at the entire set of retrotransposition-mediated duplications (not only those that have occurred in recent times, Supp Fig. 9).

### Genes with few binding TFs and without CGI in their promoters are more likely to be retained following segmental duplication

We next asked whether promoter features are associated with successful gene retention in segmental duplications. In segmental duplications, at least a fraction of the promoter region is duplicated, unlike in retrotransposition. Furthermore, genes that have duplicated as part of larger regions that have recently duplicated enable a comparison between genes that their duplicates were retained and gene duplicates that were lost. This is because we can compare the duplicated regions with syntenic non-duplicated regions in closely related species, and obtain the sets of genes that were duplicated and either subsequently lost or retained. We thus focused on a set of human genes residing within genomic regions of known recent segmental duplication, which are thought to have duplicated in the primate lineage. These regions were further filtered: (1) by excluding segmental duplications that appear as two separate regions in mouse as well, and (2) by filtering by average paralog dS in the region, to remove ancient duplications that have been lost in the mouse lineage. In addition, we carried out several more filtration stages, including the removal of triple and higher-order segmental duplications and the use of genes that their ENSEBML annotations of paralogy and orthology match our expectations, to obtain a high-confidence set of genes that have recently duplicated and either resulted in gene loss or retention (see “Methods” and Additional File 1: Fig. S10 for details). This procedure yielded comparative sets of genes that their duplication resulted in gene loss or retention in primates and, importantly, have a single gene copy in mouse, allowing for a comparison to these genes’ promoters before duplication.

**Fig. 4:**
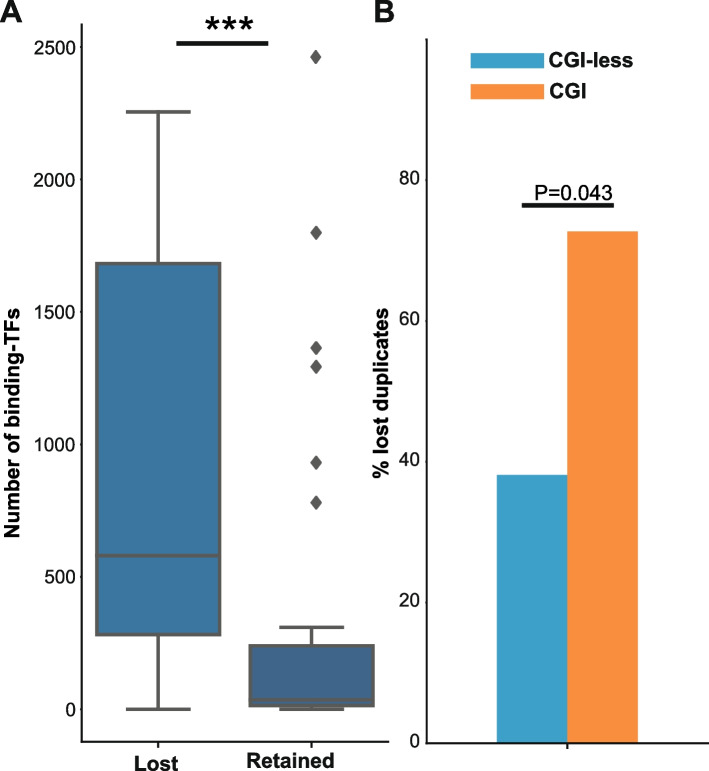
Promoter characteristics in duplicates that were lost or retained following segmental duplication in primates. **A** The number of TF binding events in promoters of mouse genes, in sets of genes that recently duplicated in primates and were either lost or retained (380 and 120 genes, respectively). Comparison between the distributions was preformed using a Mann–Whitney one-sided test. **B** The fraction of lost duplicates, out of the total number of duplicated genes that have either a CGI or CGI-less promoter architecture, in sets of genes that recently duplicated in primates (enrichment was tested using Fisher’s exact test)

For these two sets, we compared the number of TF binding events in the mouse promoter based on the Cistrome data [39]. We observe that the set of genes where both copies were retained following duplication have a significantly lower number of TF binding than those where one copy was lost (*P*-value = 8.7 × 10^−17^, Mann–Whitney test, Fig. 4A). Importantly, since gene expression level was previously suggested to be associated with gene loss [15], and since gene expression may be related to the number of TF binding events observed in ChIP-seq studies, we here show the results following regression of this potential confounder (see “Methods”). Thus, successful retention of genes following duplication is associated with a low number of TFs that bind these genes’ promoters, irrespective of gene expression.

Next, we compared how many CGI and CGI-less genes in these segmental duplication regions are either lost or retained following duplication (we define genes as CGI or CGI-less as above, and again control for gene expression as a potential confounder). We observe that CGI genes undergo a higher rate of gene loss following duplication, while CGI-less genes are more often retained (*P*-value = 0.043, Fisher’s exact test, Fig. 4B). These results suggest that promoter characteristics such as CGI presence and high number of TFs that bind it are associated with gene loss following recent segmental duplication.

### Duplication events involving CGI genes are ancient and have mostly occurred before the emergence of vertebrates

In the previous analyses, we found an enrichment of CGI-less genes to be successfully retained following recent retrotransposition-mediated duplications as well as in segmental duplications. Since CGI is a frequent element of many mammalian gene promoters, we next studied the distribution of paralogs in terms of their promoter types along an evolutionary timeline spanning from ancient duplications (that have occurred in the ancestors of Opisthokonta and Bilateria) to the most recent duplications in human and mouse genomes. For this analysis, we split paralogs to three categories—those where both copies include a CGI in their promoter (“CGI paralogs”), those where both are depleted of CGIs in their promoters (“CGI-less paralogs”), and those where a CGI is found in only one of the paralogs’ promoters (“Mixed paralogs”).

**Fig. 5:**
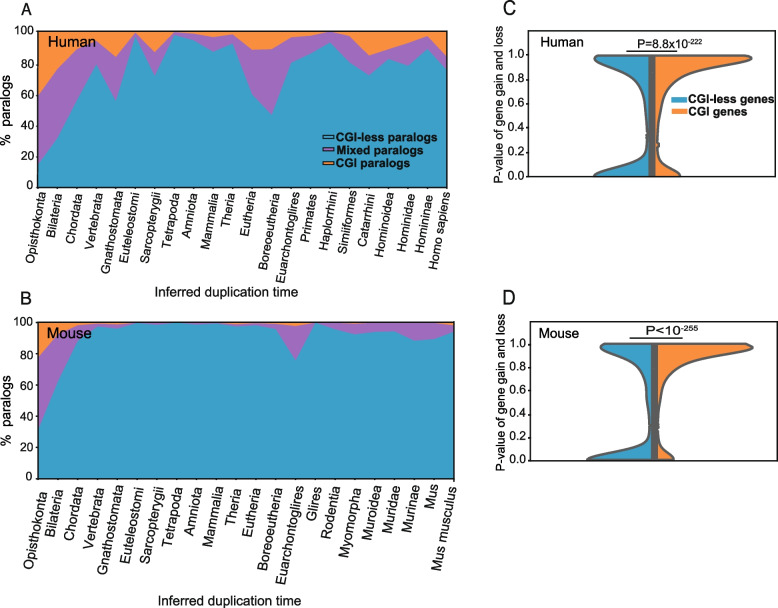
CGI and CGI-less genes duplication over evolutionary time.** A** A timeline showing the relative fractions of human paralogs at each TMRCA from Opisthokonta to Homo Sapiens, where each point is split into CGI paralogs, CGI-less paralogs, and Mixed paralogs. **B** The same as in **A**, only with mouse paralogs. CGI and Mixed pairs are skewed towards ancient times of duplication in both human and mouse paralogs (permutation test, *P*-value < 10 − 5). **C**,**D** Distributions of *P*-values of rates of gain and loss in CGI and CGI-less genes in human and mouse. Comparison between the distributions was preformed using a Mann–Whitney one-sided test. Group size numbers are as in Fig. 1A,B

We observe that those pairs that include CGI genes (either CGI paralogs or Mixed paralogs) have duplicated almost exclusively in ancient evolutionary times, before or around the time of vertebrate emergence. This is true for both human and mouse paralogs and contrasts with the enrichment of CGI-less gene duplication in recent evolutionary times (Fig. 5A,B, and Additional File 1: Fig. S11A-B, showing the relative fractions and absolute numbers of paralogs, respectively).

We next identified ohnologs—gene duplications originating from whole-genome duplication events, by implementing the OhnoDB methodology [42] and running it with the genome annotations we used (see “Methods”). In line with their ancient origins, we observe that ohnologs are enriched in CGI paralogs with respect to other paralogs that are products of small-scale duplications (*P*-value < 10^−298^, chi-squared test, Additional File 1: Fig. S12).

Thus, following the establishment of CGI as a major regulatory element in gene promoters during, or close to, the emergence of vertebrates [43], nearly all successful events of gene duplication and retention involved genes that are devoid of CGIs in their promoters. We confirmed this by comparing the rate of gene gain and loss of CGI versus CGI-less genes in human and mouse, while controlling for gene expression levels (Fig. 5C,D). We observe that CGI-less genes have higher rates of gene duplication in both genomes, which is in agreement with the previous analysis that was based on paralogs. Finally, when looking at the fraction of one-to-one orthologs between human and other vertebrates, we consistently observe a higher fraction of one-to-one orthologs in CGI genes in comparison with CGI-less genes (Additional File 1: Fig. S13). This further strengthens the notion of higher retention rates of CGI-less genes following duplication (and decreasing the fraction of one-to-one orthologs). Since CGI emerged as a regulatory element approximately at the time of vertebrate emergence, ancient duplicates predating vertebrates that have CGI in both paralogs presumably evolved the CGI elements in their promoters independently.

### CGI paralogs share greater similarity of TF binding patterns between their promoters than CGI-less paralogs

**Fig. 6:**
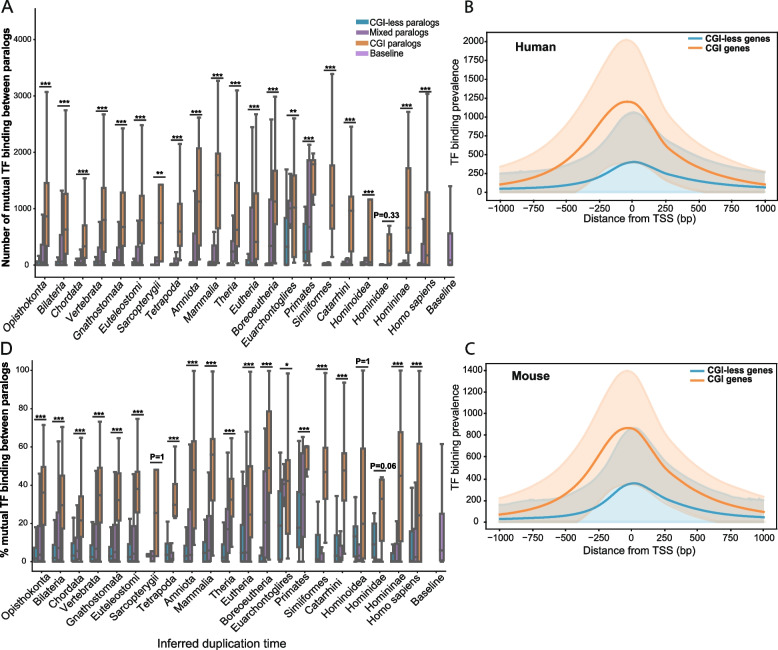
TF binding in promoters of CGI and CGI-less genes and paralogs. **A** A timeline showing the total number of TFs that bind to promoters of both paralogs. Paralogs are split based on their inferred time to most common recent ancestor (TMRCA)—from Opisthokonta to Homo Sapiens, and are further split based on their promoter classification: CGI—CGI paralogs (orange), Mixed (purple), CGI-less—CGI-less paralogs (blue). In each inferred time, CGI and CGI-less paralogs are paired by expression, to control for gene expression level. Group size numbers are as in Fig. 1A. **B** A TSS-relative histogram of TF-ChIP-seq peaks from the Cistrome dataset for CGI and CGI-less genes (10,216 and 9520 genes, respectively). The cumulative number of TF-ChIP-seq peaks from the Cistrome dataset that intersect with promoter regions of CGI and CGI-less human genes are shown. The shaded region represents one standard deviation from the mean. **C** As in **B**, only with mouse genes (6955 and 14,830 genes, respectively) and with the mouse ChIP-seq data from Cistrome. Both human and mouse CGI genes have a greater number of TF binding to their promoter regions in comparison with CGI-less genes (*P*-value < 1 × 10^−307^, *t*-test). **D** As in **A**, only in fractions—the percentage of TFs that bind to promoters of both paralogs, out of the total number of TFs that bind to the promoters of both paralogs. Comparison between the distributions of CGI paralogs and CGI-less paralogs in **A** and **D** was preformed using a Mann–Whitney one-sided test, and corrected by FDR. (***P < 0.001, **P < 0.01, *P < 0.05)

Previous studies suggested that CGI-less genes display larger dynamic range in transcription between conditions, such as before and after immune stimulation. Importantly, CGI-less genes have higher transcriptional divergence between orthologs, while orthologous CGI genes display lower transcriptional divergence and lower plasticity in expression [4, 30, 44]. We thus asked whether these differences are also reflected in paralogs—that is, if the presence and absence of CGIs in promoters of paralogs is associated with the degree of conservation of *cis*-regulation between these duplicated genes. To test this, we used the Cistrome database [39], to obtain the set of TFs that binds each gene’s promoter (calculated as described above, see “Methods” for details). Next, we calculated for each pair of paralogs the total number of mutual TFs, those TFs that bind to promoters of both paralogs. We observe that a significantly higher number of mutual TFs exist for CGI paralogs in comparison with CGI-less paralogs (Fig. 6A). This is true for both recent and ancient duplication events and is also observed in mouse genes (Additional File 1: Fig. S14A). Importantly, in all comparisons, we control for gene expression between the sets of CGI and CGI-less paralogs, to avoid the potential bias of higher gene expression of CGI genes. Interestingly, Mixed paralog pairs show intermediate levels of mutual TFs, between high numbers of mutual TFs observed in CGI paralogs and low numbers observed in CGI-less paralogs (data not shown).

The number of TFs bound to CGI genes is significantly higher than that of CGI-less genes (Fig. 6B,C). This higher number of binding TFs may bias our results when using absolute numbers of TFs, as in Fig. 6A. We thus asked whether the fractions of mutual TFs, out of the total number of TFs bound to either of the paralogs’ promoters, differ between CGI and CGI-less paralogs. We observed that the fraction of mutual TFs is significantly higher in CGI paralogs, and this is observed in both human and mouse, regardless of duplication time (Fig. 6D and Additional File 1: Fig. S14B). These analyses suggest that *cis*-regulation, in terms of the specific set of TFs that bind gene promoters, is significantly more conserved between CGI paralogs, in agreement with the notion that CGI genes are less plastic in their transcription. Interestingly, this is consistently observed across ancient and more recent duplications, although the fraction of CGI paralogs is significantly smaller in recent duplication events. Finally, the observed results are controlled for gene expression level, thus the higher conservation in *cis*-regulation of CGI paralogs is not due to gene expression.

When looking at ancient gene duplicates with relatively high numbers of shared TF binding between the two paralogs (above 50%) we observe, using g:Profiler [45], that the genes of such duplicates are enriched in pathways relevant to basic cellular pathways (e.g., Protein processing in endoplasmic reticulum) and signaling (e.g., Beta-catenin phosphorylation cascade). Thus, ancient duplicates that are regulated by a similar set of TFs are often involved in basic intracellular processes, as opposed to ancient gene duplication that involve major shifts in gene expression such as tissue-specific genes. See the full list of genes and enriched terms in Additional File 2: Table 4A-D.

## Discussion

Gene gain and loss are thought to be major processes that shape genome novelty and species adaptation [46]. While numerous studies focused on the evolution of gene duplicates themselves, from the level of sequence and transcriptional divergence between paralog genes, to how new genes are incorporated into existing protein complexes and networks, relatively few works studied the evolution of non-coding regions that regulate gene duplicates [10, 19, 21, 24, 25, 47–49]. In this work we focused on the evolution of promoters of gene duplicates, on *cis*-regulatory regions in newly duplicated genes, and on the association between different promoter characteristics with the fate of these duplicates. We performed analyses to profile and compare paralogs’ promoters at the level of sequence, *cis*-regulation, and overall architecture.

We first analyzed promoter sequence similarity between paralogs, partitioned by their duplication time and by their duplication mechanism. We observe that with the exception of recent duplication in primates or rodents, there is little to no significant similarity between paralogs’ promoter sequences. This trend is observed regardless of the promoter length we use in the comparison. Given sufficient time since duplication, promoters of the two duplicates usually accumulate mutations and undergo different evolutionary processes such that their sequences show no higher similarity than the observed sequence similarity between promoters of unrelated genes. The few paralog genes that have duplicated in ancient times and that their promoter sequences are relatively conserved are enriched in particular gene classes, including certain histones, keratin proteins, olfactory receptors, and zinc finger proteins.

Previous work studying signatures of selection in gene promoters, suggested an enrichment of positive selection in promoters of gene duplication [29], which is in line with our results and may explain some of the observations we made regarding rapid changes between promoters of gene duplicates. In both human and mouse, we observe that a fraction of recently duplicated paralogs displays relatively high sequence similarity between their promoters and that this similarity is reduced with time from duplications. We note that the observed similarity between promoter regions of recent duplicates may stem from nonallelic gene conversion, similar to previous observations in gene bodies of recent duplicates [50, 51]. Finally, this relatively high sequence similarity in some of the recent duplicates is only observed in paralogs that are inferred to have duplicated through segmental duplication rather than retrotransposition-mediated duplication. This is expected given the different copying mechanisms, where duplication of segments may include parts of the promoters, while retrotransposed genes are copied without their promoters.

Retrotransposed gene copies often reside in new chromatin environments that are dissimilar to the corresponding region upstream of the original gene [52–54]. This is in agreement with the observed low sequence similarity between retrotransposition-mediated duplicates. Thus, in these cases, a functional promoter must either evolve de novo or be recruited from existing regulatory elements in the vicinity of the retrotransposed gene [53]. Our findings of a relatively low number of TFs that bind the promoters of recently duplicated retrocopies (in comparison with the number of TFs bound to the original gene’s promoter) strengthen this notion. Furthermore, this finding of a low number of binding TFs, as well as the observed low cross-tissue expression of new retrocopies, agrees with a previous study that suggested an age-dependent acquisition of active histone modifications to retrocopied genes [54]. Additionally, we observe clear trends of the propensity for specific promoter architectures to occur in promoters of the original and the retrocopy genes, suggesting that local chromatin constraints can influence duplicated gene evolution. In this respect, we observe that while the original gene may have a CGI in its promoters, the retrocopy gene promoter is almost always depleted of such CGI elements. This may point to greater constraints imposed on regulation through CGIs, precluding events of retrotransposition near CGI regions to be successfully retained. Alternatively, this depletion of CGIs in promoters of recent retrogene copies can be associated with the evolutionary time required to acquire such a CGI element as part of a functional promoter.

Our analysis of gene duplications in recent segmental duplication regions allows an investigation of various factors that affect the fate of gene duplicates in these regions. By mapping cases of gene duplicates that were lost or retained within this region, we were able to study how promoter characteristics are associated with gene loss or retention. We observe that a lower number of TFs that bind to the promoter and the absence of CGIs are both associated with gene retention following duplication. These findings underscore how promoter characteristics may impact transcriptional evolvability of new gene copies and subsequently their fates. Importantly, in our analysis, we controlled for gene expression level, which was previously associated with gene retention and loss [15]. Thus, our findings show that promoter characteristics are important in determining gene fate following duplication.

This is further demonstrated in our analysis of the rate of gene duplication of CGI and CGI-less genes in human and mouse. In both species, we observe a higher rate of duplication in CGI-less genes. When profiling paralogs according to their promoter architecture, we observed that gene duplications that include at least one CGI gene are usually ancient and that most recent duplication events in mammals involve CGI-less genes. This distinction between ancient and recent duplicates is in agreement with previous findings regarding the differences in functions of ancient and young paralogs [17, 18]. Importantly, our promoter-based analysis also points to an additional important characteristic of recent gene duplicates: CGI promoters are associated with greater robustness in expression of the genes they regulate. In contrast, CGI-less genes tend to have greater dynamic range in their transcription and display a higher transcriptional divergence between orthologous genes [4, 36, 44, 55]. Thus, CGI-less promoters in recent gene duplicates may facilitate transcriptional divergence between the duplicates and enable faster gene neo- or sub-functionalization. This is in line with our observations that the number and fraction of mutual TFs that bind to both paralog promoters is significantly lower in CGI-less paralogs in comparison with CGI paralogs. It is also in agreement with a previous study of histone mark conservation across mammals that suggested that GC content is associated with conservation of promoter activity [56]. The depletion of CGI genes and genes with relatively large number of TF bindings in their promoters in retained gene duplicates can also be explained by the fact that incorporation of such highly expressed genes, or genes with complex regulation, into the gene network may lead to deleterious effects, as suggested by previous work analyzing gene expression of gene duplicates [15, 23]. Thus, CGI-less genes and genes with few TFs regulating their expression may be initially integrated into the network more easily and subsequently diverge more rapidly in expression, supporting their preservation as two distinct duplicates.

We note that promoter architecture that lacks CGIs is also associated with nosier and less homogenous gene expression, as observed in single-cell RNA-seq studies [4]. CGI promoters counterintuitively also show decreased sequence conservation across orthologs [4, 44]. This leads to seemingly opposite characteristics of CGI-regulated genes: Their expression is more conserved and robust across species and conditions than CGI-less genes, but their promoter sequences are less conserved. This could be explained by greater tolerance to mutations of CGI promoters that lead on the one hand to higher accumulation of mutations in CGI promoters but on the other hand supports homogenous and conserved gene expression of CGI genes.

Thus, recent gene duplicates with CGI-less promoters may be transcribed in a noisy manner. These characteristics are suitable for the function and regulation of certain genes, including cytokines and chemokines, an important class of immune-related genes that display high transcriptional range and high cell-to-cell variability in expression [4]. Indeed, many cytokine and chemokine families have been shown to also undergo rapid gene gain and loss in the course of mammalian evolution [4].

## Conclusions

In summary, our work provides a detailed characterization of the divergence of promoters of duplicated genes from ancient to recent duplication times, at both the sequence and the *cis*-regulatory levels. In particular, our study of recent small-scale duplication events in mammalian genomes demonstrates how different promoter characteristics are associated with loss and retention of new gene duplicates. These results underscore the importance of *cis*-regulatory mechanisms in shaping the evolution of new genes and their fate following duplication.

## Methods

### Gene and paralog annotations

We downloaded gene annotations, including orthology and paralogy assignments, from ENSEMBL version 98, corresponding to GRCh38 and GRCm38 genome assemblies for human and mouse, respectively. We removed genes that are not protein coding or whose transcripts are not known, and kept only the primary assembly genes. Similarly, for pairs of paralogous genes, we only included pairs of genes where both genes are coding, resulting in a total of 133,328 pairs of paralogs in humans and 356,568 pairs in mouse. We note that the majority of annotated pseudogenes are not part of the annotated paralogy dataset in ENSEMBL. Only polymorphic pseudogenes (that are partially active in a fraction of the human population) are part of the paralog dataset and they constitute a small portion of it (2003 pairs of coding genes—polymorphic pseudogenes).

We separated paralogs based on their inferred time of duplication based on two different and commonly used methods [14, 23, 37]: (1) Inferred duplication time based on ENSEMBL tree and provided by ENSEMBL Compara [37], and (2) a molecular clock approach based on the synonymous substitution rate—dS, between the two paralogs in their coding sequences (where higher dS values imply longer time since duplication [23, 25]). For the latter method, we binned paralog pairs into 24 bins: Paralogs that significantly diverged in coding sequence (i.e., with high dS values, above 2) were binned into a single bin, which likely includes many ancient categories of paralogs. This resulted in the first bin being much larger than the other 23 bins. In addition, all zero-value dS paralogs were binned into the 24th bin, which is likely enriched with recent duplications. This resulted in an equal size for all bins except for the first and the last bins that are larger than the rest.

Both methods (a molecular clock approach and a tree-based approach) have been used in previous studies to estimate the time of duplication [14, 23, 37]. The resulting age distribution of paralog pairs across the studied evolutionary timeline is largely in agreement with previous analysis, including the high number of ancient paralogs and the observed differences between the human and the mouse clade [17].

For each gene, we obtained the rate of its gene family expansion and contraction, as computed using the CAFE algorithm [57], from ENSEMBL Compara [37].

To test our analysis with a non-redundant set of paralog pairs, we subset each gene family to N-1 paralog pairs (each gene family of N paralogs is a result of at least N-1 duplication events).

We chose N-1 paralog pairs by taking adjacent genes in the gene tree (from the ENSEMBL gene tree), when walking across the gene tree in a DFS (Depth First Search) order. This subset is likely to be enriched with direct results of duplication events.

### Promoter sequence similarity analysis

Promoter sequence similarity between paralog genes was evaluated by performing local alignment, using the function pairwise2.align.localds from biopython, on the segment 300 bp upstream of the TSS. TSS coordinates were taken for each gene from the canonical transcript (defined as the longest transcript among the gene’s transcripts, which has the best TSL value (transcript support level), or the longest transcript if no TSL transcripts exists) for each gene. We were careful not to take any base pairs downstream of the TSS, since those are expected to be more conserved, being part of the gene body itself, and would bias the results.

To estimate local alignment, we used a nucleotide similarity matrix between the two paralogs’ promoter regions, based on Kimura’s 2-parameter model (K2P) [38] with transition counting as − 1 and transversion as − 2, while matching bases counting as + 4 and gaps as − 2. The similarity score per paralog pair is the cumulative values of the matches of their nucleotides across the promoter region. The definition of promoter regions can differ depending on gene and analysis [30, 58, 59]. We thus repeated the promoter sequence similarity analysis with promoter length of 100, 500, and 1000 bp upstream of the TSS (in addition to 300 bp). Thus, for every pair of paralogs, the cumulative matching values along the promoter region (either 100, 300, 500, or 1000 bp) were obtained as a similarity measure.

We note that for the majority of this work, we focus on promoter regions of 300 bp upstream of the TSS, based on previous works that used this definition for related analyses on promoter characterization across orthologs [4, 44].

To obtain the baseline level of promoter sequence similarity that is achieved by “random,” we compared promoter sequences of all pairs of randomly selected 10,000 genes in either human and mouse. This baseline calculation allows to compute the distribution of similarity sequences between unrelated promoters, to enable a quantification of which “real” paralog pairs have a significantly higher similarity than random. The average baseline similarity score is 51 and 54 in mouse and human promoters.

### Functional enrichment analysis

We used gProfiler [45] to find enriched pathways within each subset of paralogs that have (1) an exceptionally conserved promoter sequences and (2) very high levels of shared TF bindings between the two duplicates. This enrichment was done with default settings (i.e., against the background of all genes). The results of the significantly enriched functions (FDR-corrected *P*-values < 0.05) are shown in Additional File 2: Tables 3–4.

### Inference of duplication mechanism

Recent gene duplications in human and mouse can be divided by duplication mechanism into segmental and tandem duplications (denoted as “segmental” hereafter), and to retrotransposition-mediated duplications. Segmental and retrotransposition-mediated duplications were determined using a previously described method [23] with several modifications (such as excluding pairs that one or both copies lacked any UTR). Briefly, retrotransposition-mediated duplications were identified by finding paralogs where one copy (the original copy) has more than two exons, while the retrotransposed copy (“retrocopy”) has only one exon. From the remaining paralogs, pairs were considered as “segmental duplications” if at least 80% of exon junctions (at least two such junctions) between canonical transcripts were congruent. That is, their distance in the pair alignment of the transcripts was no more than 10 bp.

We note that this analysis of distinguishing between duplication mechanisms based on comparison of gene structure between paralogs is most accurate in relatively recent gene duplications, and we thus focus much of the analysis on such recent duplications. This is also true for the identification of the original copy and the retrotransposition-mediated copy (“retrocopy”), in the case of retrotranspositions. We also note that a fraction of the annotated paralogs in ENSEMBL were not included in either the segmental or the retrotransposition sets, since their gene structures did not match any of the abovementioned criteria.

Ohnologs (paralogs resulting from ancient whole-genome duplication events) were identified using OhnoDB methodology [42], but recomputed based on ENSEMBL version 98, since the phylogenetic tree from ENSEMBL, used to infer the age of paralogs, was substantially modified and expanded between the ENSEMBL versions used for the original OhnoDB 2.0 and ENSEMBL version 98.

### Transcription factor binding analysis

ChIP-seq data (in the format of narrowPeak bed files) of a large set of human and mouse transcription factors binding experiments were downloaded from the Cistrome database [39]. From this data, we excluded a few general factors and insulators: CTCF, RAD21, REST, EP300, and RNA Polymerase (POLR), and removed TFs with no binding recorded. With the filtered TF binding data, we counted for each of the human and mouse genes which and how many TFs intersect in their binding region with the gene’s promoter area. This was done by testing the overlap of at least one base pair between the promoter region and the peaks in the narrowPeak bed files.

In Fig. 6B,C, we examined the total number of TF binding events for each CGI and CGI-less genes, in a region of 2000 bp up- and downstream of the TSS of each gene. We note that for CGI gene determination, we use a region of 300 bp upstream and 100 bp downstream of the TSS that is thought to represent the likely region where CGI elements relevant to regulation of the gene will be located, while avoiding incorporation CGI elements that are unrelated to the gene in question.

In Fig. 6A, D, we compared the similarity of TF binding to promoters of pairs of paralogs, by looking at (1) the total number of shared TF bindings and (2) the fraction of the shared TF bindings from the total number of TF binding events, in the promoters of the two paralogs. Thus, for each paralog pair, we computed the total number and relative fraction of TF bindings out of all binding events that are shared between the two genes.

When comparing the similarity in TF binding, either in absolute numbers or in relative fractions, we controlled for gene expression, by pairing genes based on similar average gene expression across tissues between the groups of CGI and CGI-less paralogs that have duplicated at the same evolutionary period (see below).

### CGI and promoter classification

For the human genome, CGI annotations were downloaded from ENSEMBL as well as from UCSC Genome browser [60] [https://genome.ucsc.edu/cgi-bin/hgTables] (Assembly 2013/12 hg38). These annotations gave nearly identical results (see Additional File 1: Fig. S15). We show all analyses using the UCSC Genome browser annotations. Since CGI predictions in species other than human were shown to diverge from experimental data of non-methylated regions [61], we used ENSEMBL CGI annotations for mouse (as ENSEMBL also includes experimental data).

We defined CGI genes—genes harboring CGIs in their promoters—as genes that at least 50% of the region spanning from 300 bp upstream of the TSS and 100 bp downstream of it overlaps with annotated CGIs, as previously done [4, 44]. All other genes were defined as CGI-less genes. We note that the set of CGI genes based on these promoter definitions is largely in agreement with other promoter length used (see Additional File 1: Fig. S16).

These CGI definitions for each gene, allow us to define three groups of paralogs, based on their CGI status: (1) CGI paralogs—where both paralogs are CGI genes, (2) CGI-less paralogs—where both paralogs are CGI-less genes, and (3) Mixed paralog pairs—where only one of the genes is a CGI gene. These definitions are mostly relevant for the analyses shown in Figs. 5A–B and 6A, D.

When comparing the rate of gene gain and loss between CGI and CGI-less genes (Fig. 5C,D), we control for gene expression using a similar approach to comparison of CGI and CGI-less duplications in segmental duplications. Briefly, we used only paired genes (a CGI gene paired with a CGI-less gene), such that they display similar levels of expression across tissues (log(TPM) difference in the pair could not exceed 0.1 log(TPM)). Gene expression across tissues was obtained from GTEx and BodyMap, for human and mouse, respectively (as described below).

### Gene expression analysis

To obtain expression levels of studied genes across a large set of tissues, we used RNA-seq data from the Genotype-Tissue Expression (GTEx) project, version 8 [40]—a large transcriptomics dataset with gene expression across different tissues from a large number of human individuals. We filtered out all the pseudoautosomal expression records, along with non-primary tissues (cultured cells, EBV-transformed lymphocytes and CML). For all expression-based analyses, we followed the same filtering used in Lan et al. [23], by removing any genes with total expression below 5 TPMs across all tissues as well as those whose expression levels are below 0.5 TPM in every single tissue. For mouse gene expression, we used the BodyMap dataset [41], and performed similar filtering as described for human.

### Analysis of loss and retention in segmental duplications

In this analysis, we aimed to contrast cases of gene duplications where the duplicates were retained with those cases where one of the duplicates was lost, to study how these two scenarios (of retention versus lost) may differ in their promoter characteristics. For this, we required a set of genes where we have high confidence that a recent duplication has occurred and was either followed by retention or loss. Most standard methods cannot distinguish between loss and lack of duplication; we thus needed to develop an approach to obtain a set of genes where a duplication followed by loss has occurred. We required the duplications to be relatively recent, since in more ancient duplications many changes in the chromosome structure are likely to have occurred and these can mask the signal and make the results more difficult to interpret. Thus, we chose to use annotated regions of segmental duplications in human, where we can find both groups of recent duplication events with the ability to compare between them and to study their promoter regions (see Additional File 1: Fig. S10).

For this, annotated segmental duplication regions in human were retrieved from the UCSC Genome Browser (GRCh38/hg38 database) [60]. For each duplicate segment, we tried to map both segments onto the mouse genome using liftover [62], to obtain a syntenic region. If any of the segments required a split (i.e., it did not map to a single syntenic region by liftover), we excluded that segmental duplication region from the analysis. If both segments mapped close enough (> 50% overlap) to the same mouse syntenic region, we proceeded with them to the next filtering stage. If one of the human segments did not map at all, we assumed it was a novel region and proceeded to the next stage. The previously described stages were performed to filter ancient duplications—those that predate the split between rodents and primates. Next, we also filtered triple and higher-order segmental duplications. In addition, we filtered cases that were suspected to be an ancient segmental duplication with a subsequent event of deletion in rodents: For this, we scanned all paralogs, as defined by ENSEMBL, between the segmental duplications of the human genome and averaged their dS (rate of synonymous substitutions). We compared this value to the average dS value for paralogs inferred to have duplicated at the branch point of Euarchontoglires: If the average dS value was larger than half of this value, we considered this segmental duplicate to have occurred before the primate-rodent split (followed by a recent loss event in the branch leading to mouse) and excluded it.

With the remaining segmental duplications in human, we carried out a global alignment procedure between the three gene sequences (on the mouse segment along the strand and on both human segments along their respective strands). We considered genes to be “equivalent” if they have a homology relationship defined in ENSEMBL (orthologs between mouse and human or paralogs between two human genes). We then divided the remaining gene set into those where the duplicated genes were either both retained or one of them was lost, as follows: We tested which genes are present on the mouse segment and on one of human segments, but not the other human segment—those are inferred to be recent gene losses in human. In contrast, gene homologs present on all three segments are inferred to be retained gene duplicates in human, following segmental duplications.

We controlled the quantification of relative fractions of loss and retention for average gene expression levels (see below) since it has been previously shown that gene expression can affect duplicate gene retention [15]. This was achieved by pairing sets of retained genes with sets of lost genes, by using the average expression of the mouse gene belonging to each (this was done since in the entire test of tested genes, we always have one gene copy in mouse)—the log(TPM) difference in the tested pair could not exceed 0.8. log(TPM). TPM values were based on mean gene expression across tissues, from the mouse BodyMap dataset [41].

For analyzing the association between numbers of TF binding events in promoters and gene retention or lost, we counted the number of TF bindings events in the mouse promoters for the set of gene duplicates where both copies were retained following segmental duplication in primates, and the same for the set of gene duplicates where one copy was lost.

When analyzing fraction of gene loss in segmental duplications with genes with and without CGI in their promoters, we used the promoter status in the mouse gene to determine the gene “CGI status.” We note that “CGI status” should largely remain conserved between species as closely related as human and mouse [61].

### Statistical analysis

Statistical tests (*t*-test, Mann–Whitney, Fisher’s exact, chi-squared test, and FDR correction) were preformed using either the SciPy package version 1.5.3 [63] or using R (version 4.0.5).

## Supplementary Information

**Additional file 1:** **Fig S1.** Sequence similarity between promoters of paralogsin human with various promoter sequence lengths. **Fig S2.** Sequence similarity between promoters of paralogs in mouse with various promoter sequence lengths. **Fig S3.** Sequence similarity between promoters of paralogs in human with a reduced number of paralogs. **Fig S4.** Sequence similarity between promoters of paralogs in mouse with a reduced number of paralogs. **Fig S5.** Sequence similarity between promoters of paralogs in human and mouse genomes and human-mouse orthologs. **Fig S6.** Sequence similarity between promoters of human paralogs in segmental *versus* retrotransposition-mediated duplications, with a reduced number of paralogs and with various promoter lengths. **Fig S7.** Sequence similarity between promoters of mouse paralogs in segmental *versus* retrotransposition-mediated duplications, with a reduced number of paralogs and with various promoter lengths. **FigS8.** Percentage of gene duplicates that reside in the same chromosome. **Fig S9.** Partition of retrotransposition-mediated duplications by promoter architecture. **Fig S10.** Work flow to obtain comparable sets of gene duplicates that were retained or lost in human segmental duplication regions. **Fig S11.** CGI and CGI-less genes duplication over evolutionary time. **Fig S12.** Distribution of CGI and CGI-less duplicates in different types of duplication mechanisms. **Fig S13.** CGI and CGI-less geneduplication across species. **Fig S14.** TF-binding in promoters of CGI and CGI-less genes and paralogs. **Fig S15.** Agreement of CGI annotations between UCSC and ENSEMBL. **Fig S16.** Agreement of CGI gene definitions when using different promoter length definitions to overlap with CGI regions.

**Additional file 2:** **Table 1.** Details of group numbers for Figure 1. **Table 2.** Details of group numbers for Figure 2. **Table 3.** Lists of human and mouse genes from ancient duplications with high level of promoter sequence similarities and their functional enrichment. **Table 4.** Lists of human and mouse genes from ancient duplications with high level of TF-binding similarities and their functional enrichment**.** **Table 5.** Assembled raw data of genes and paralogs.

## Data Availability

Scripts developed for this analysis can be found in: https://github.com/EFraim/Promoter-evolution-of-mammalian-gene-duplicates[64]. Major datasets used and assembled for this work can be found in Additional File 2: Tables 5A-D.

## Abbreviations

K2P: Kimura’s 2-parameter model
TSS: Transcription start site
CGI: CpG Island
TF: Transcription factor
TSL: Transcript support level
