# Supplementary material for: Promoter evolution of mammalian gene duplicates
Source: BMC Biol. 2023 Apr 13;21:80. doi: 10.1186/s12915-023-01590-6 (PMC10100218; doi:10.1186/s12915-023-01590-6)
Supplement: Supplementary file 1 — Additional file 1: Fig S1. Sequence similarity between promoters of paralogsin human with various promoter sequence lengths. Fig S2. Sequence similarity between promoters of paralogs in mouse with various promoter sequence lengths. Fig S3. Sequence similarity between promoters of paralogs in human with a reduced number of paralogs. Fig S4. Sequence similarity between promoters of paralogs in mouse with a reduced number of paralogs. Fig S5. Sequence similarity between promoters of paralogs in human and mouse genomes and human-mouse orthologs. Fig S6. Sequence similarity between promoters of human paralogs in segmental versus retrotransposition-mediated duplications, with a reduced number of paralogs and with various promoter lengths. Fig S7. Sequence similarity between promoters of mouse paralogs in segmental versus retrotransposition-mediated duplications, with a reduced number of paralogs and with various promoter lengths. FigS8. Percentage of gene duplicates that reside in the same chromosome. Fig S9. Partition of retrotransposition-mediated duplications by promoter architecture. Fig S10. Work flow to obtain comparable sets of gene duplicates that were retained or lost in human segmental duplication regions. Fig S11. CGI and CGI-less genes duplication over evolutionary time. Fig S12. Distribution of CGI and CGI-less duplicates in different types of duplication mechanisms. Fig S13. CGI and CGI-less geneduplication across species. Fig S14. TF-binding in promoters of CGI and CGI-less genes and paralogs. Fig S15. Agreement of CGI annotations between UCSC and ENSEMBL. Fig S16. Agreement of CGI gene definitions when using different promoter length definitions to overlap with CGI regions. [file 12915_2023_1590_MOESM1_ESM.pdf]

Promoter evolution of mammalian gene duplicates

Evgeny Fraimovitch and Tzachi Hagai

Supplementary Materials:
Supplementary Figures 1-16

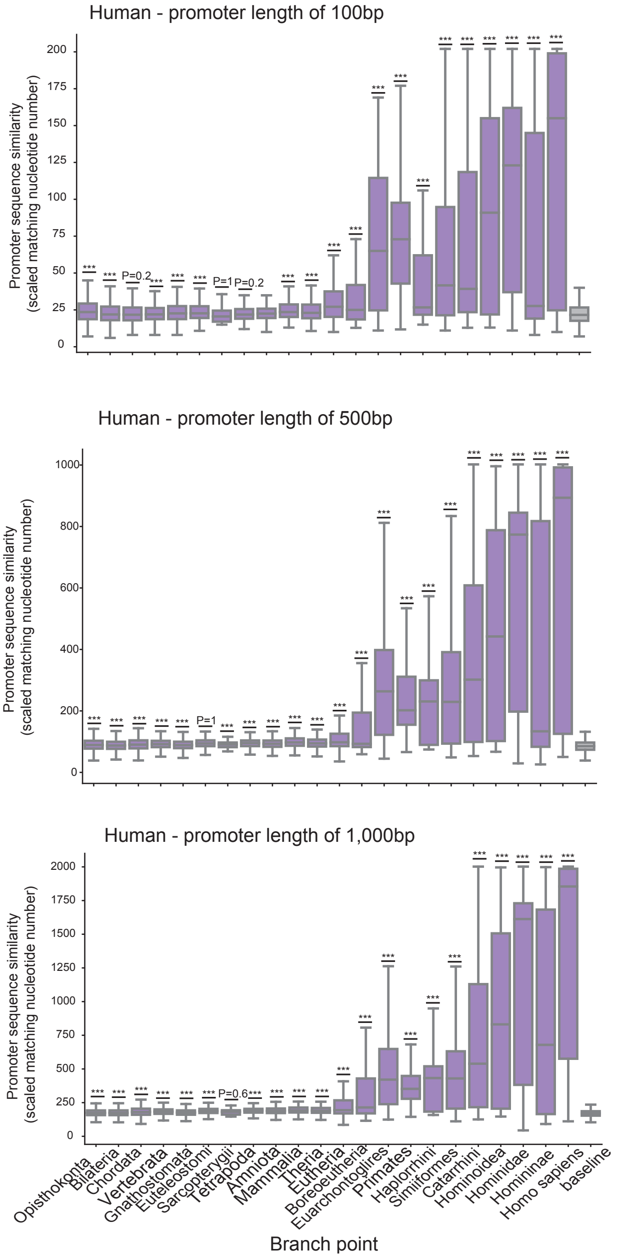

Supporting Figure 1: Sequence similarity between promoters of paralogs in human with various promoter sequence lengths.

Sequence similarity scores between promoter regions of human paralogs. In each panel the sequence length differs – 100, 500, 1,000 bp upstream of the TSS (in Fig 1A, the same analysis is shown with 300bp). Paralogs are partitioned based on their inferred duplication time (at each TMRCA (time to most recent common ancestor) from Opisthokonta to Homo Sapiens). In each group of paralogs belonging to the same TMRCA, the distribution of similarity scores is compared to that of randomly matched set of human gene promoters. Comparison between the distributions was preformed using a Mann-Whitney one-sided test and corrected by FDR. Group sizes are as in Fig 1A.

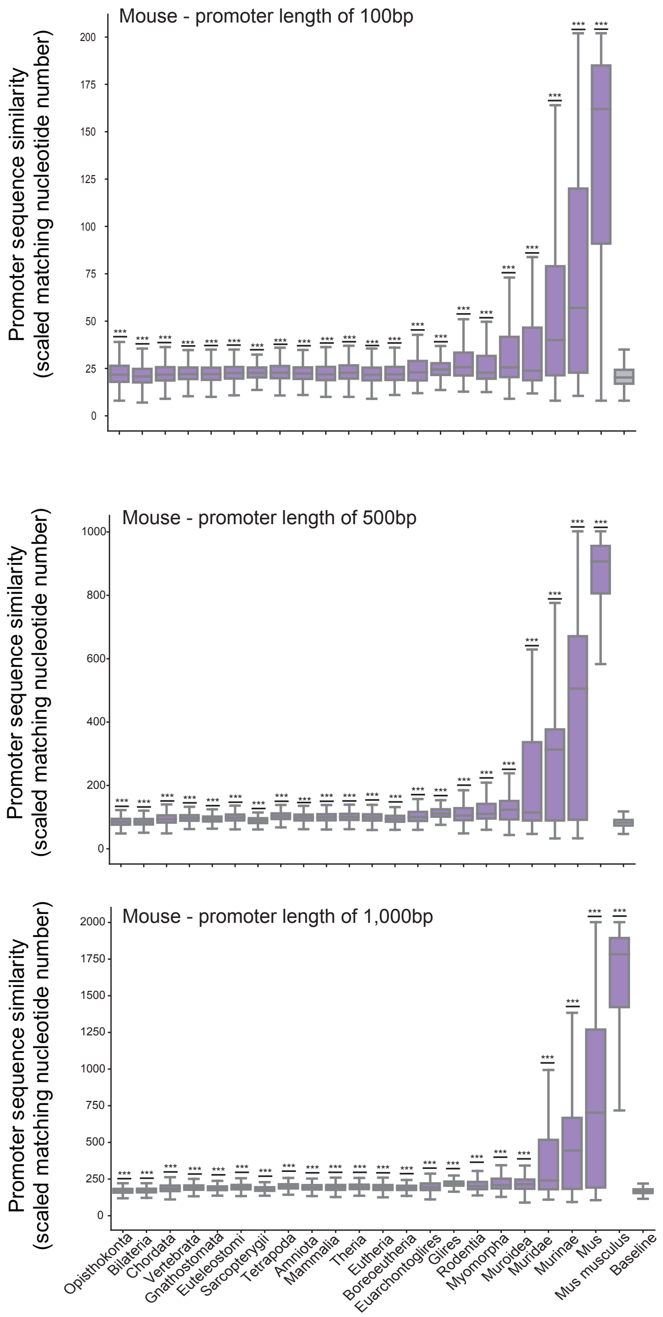

Supporting Figure 2: Sequence similarity between promoters of paralogs in mouse with various promoter sequence lengths.

Sequence similarity scores between promoter regions of mouse paralogs. In each panel the sequence length differs – 100, 500, 1,000 bp upstream of the TSS (in Fig 1B, the same analysis is shown with 300bp). Paralogs are partitioned based on their inferred duplication time (at each TMRCA (time to most recent common ancestor) from Opisthokonta to Mus musculus). In each group of paralogs belonging to the same TMRCA, the distribution of similarity scores is compared to that of randomly matched set of human gene promoters. Comparison between the distributions was preformed using a Mann-Whitney one-sided test and corrected by FDR. Group sizes are as in Fig 1B.

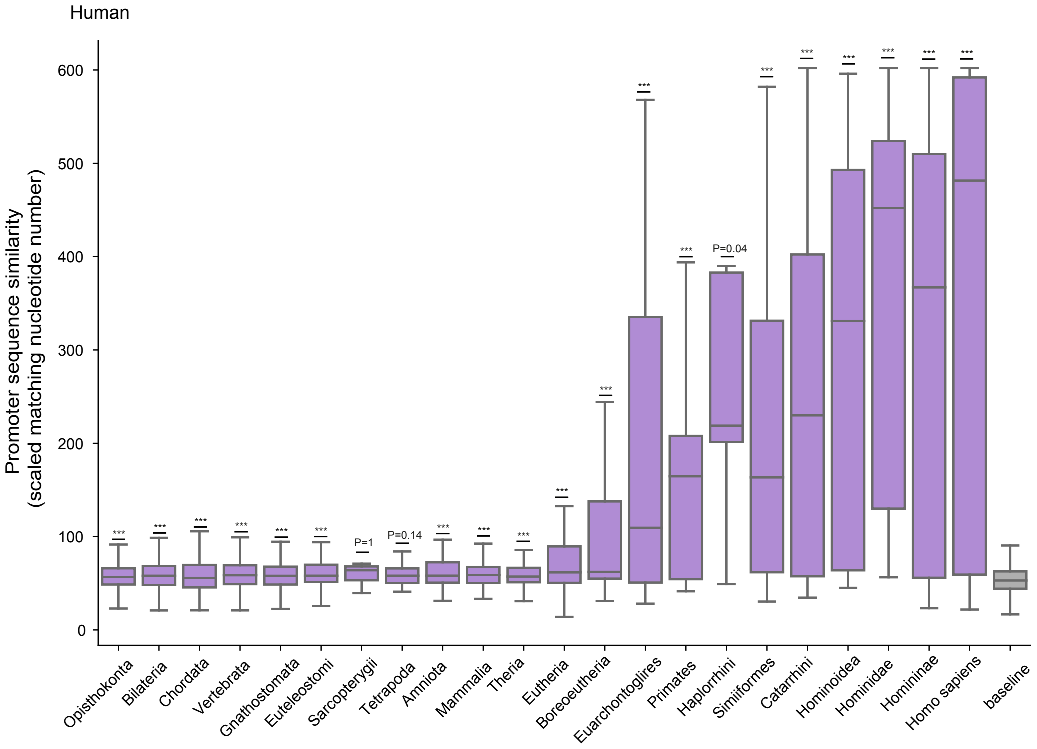

Supporting Figure 3: Sequence similarity between promoters of paralogs in human with a reduced number of paralogs.

Sequence similarity scores between promoter regions of human paralogs. As in Fig 1A, but with fewer paralogs – 6,473 pairs (N-1 pairs per a family of N gene members). Paralogs are partitioned based on their inferred duplication time (at each TMRCA (time to most recent common ancestor) from Opisthokonta to Homo Sapiens). In each group of paralogs belonging to the same TMRCA, the distribution of similarity scores is compared to that of randomly matched set of human gene promoters. Comparison between the distributions was preformed using a Mann-Whitney one-sided test and corrected by FDR.

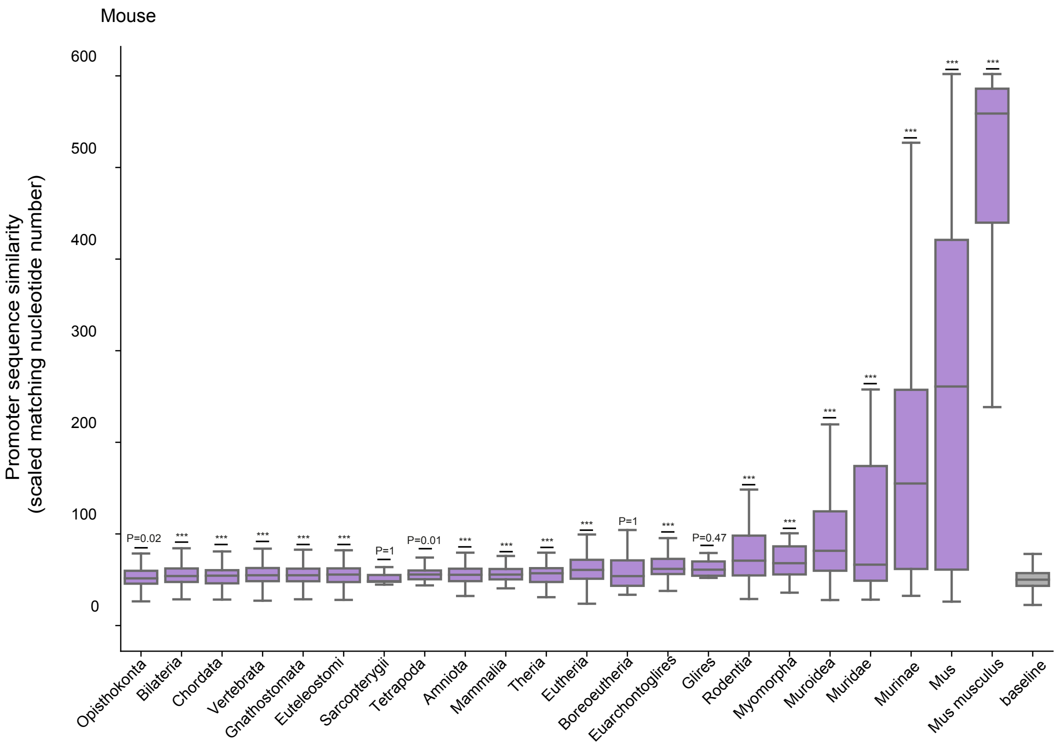

Supporting Figure 4: Sequence similarity between promoters of paralogs in mouse with a reduced number of paralogs.

Sequence similarity scores between promoter regions of mouse paralogs. As in Fig 1B, but with fewer paralogs – 6,436 pairs (N-1 pairs per a family of N gene members). Paralogs are partitioned based on their inferred duplication time (at each TMRCA (time to most recent common ancestor) from Opisthokonta to Mus musculus). In each group of paralogs belonging to the same TMRCA, the distribution of similarity scores is compared to that of randomly matched set of human gene promoters. Comparison between the distributions was preformed using a Mann-Whitney one-sided test and corrected by FDR.

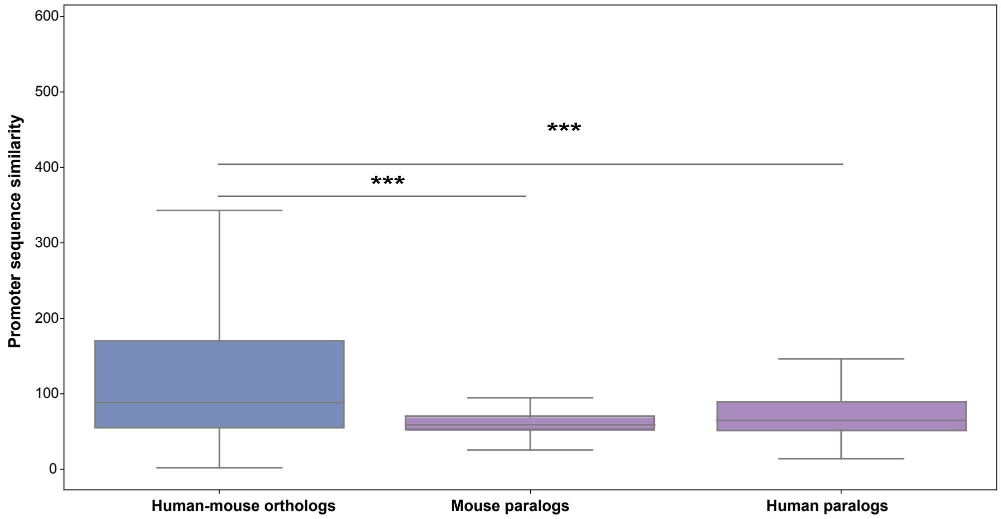

Supporting Figure 5: Sequence similarity between promoters of paralogs in human and mouse genomes and human-mouse orthologs.

Sequence similarity score between promotor regions of human paralogs and mouse paralogs that are predicted to have duplicated in the last common ancestor of primates and rodents and one-to-one orthologs between human and mouse. Comparison between the distributions was preformed using a one-sided Mann-Whitney test and corrected by FDR.

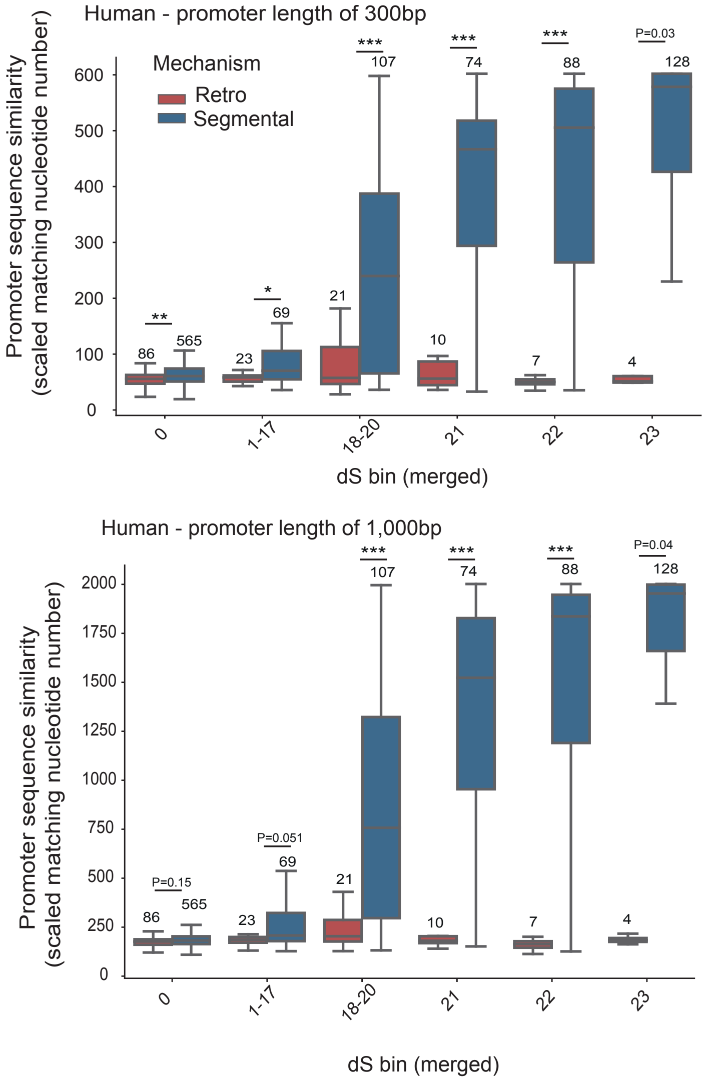

Supporting Figure 6: Sequence similarity between promoters of human paralogs in segmental *versus* retrotransposition-mediated duplications, with a reduced number of paralogs and with various promoter lengths. Sequence similarity score between promoter regions of human paralogs, where paralogs are partitioned based on their inferred duplication time (based on their dS values) and based on inferred duplication mechanism: retrotransposition or segmental duplication, in red and blue, respectively. Paralogs shown are a subset of N-1 pairs per a gene family with N members (see Methods). Top: promoter length of 300bp, bottom: 1,000bp.

Comparison between the distributions was preformed using a Mann-Whitney one-sided test and corrected by FDR. Paralogs are binned into equal-sized bins. Left-most bins represent the highest dS values, and likely the oldest duplicates, while right-most bins represent lowest dS values. (***P < 0.001, **P < 0.01, *P < 0.05). Group size numbers appear above each box.

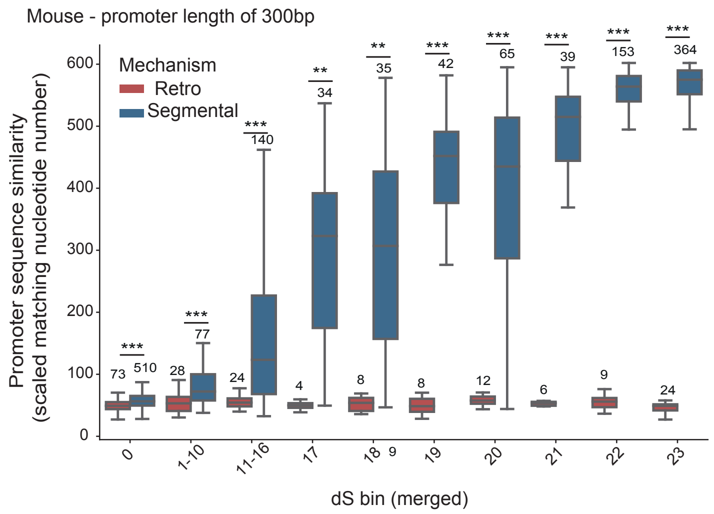

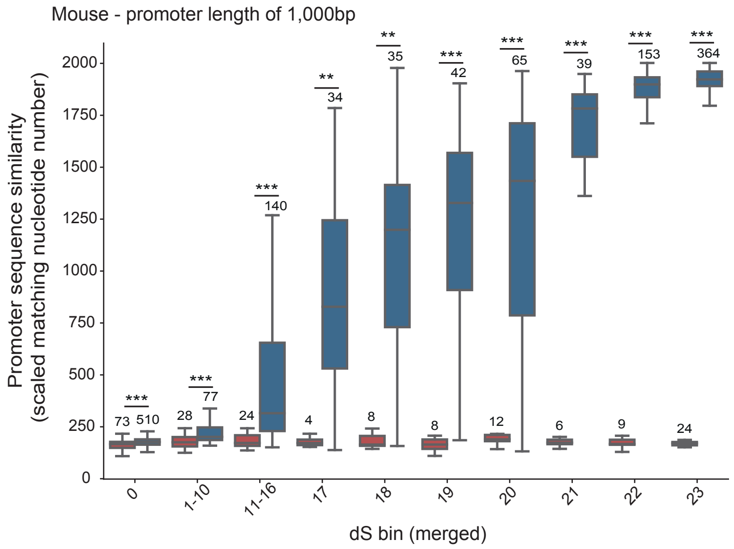

Supporting Figure 7: Sequence similarity between promoters of mouse paralogs in segmental *versus* retrotransposition-mediated duplications, with a reduced number of paralogs and with various promoter lengths. Sequence similarity score between promoter regions of mouse paralogs, where paralogs are partitioned based on their inferred duplication time (based on their dS values) and based on inferred duplication mechanism: retrotransposition or segmental duplication, in red and blue, respectively. Paralogs shown are a subset of N-1 pairs per a gene family with N members (see Methods). Top: promoter length of 300bp, bottom: 1,000bp.

Comparison between the distributions was preformed using a Mann-Whitney one-sided test and corrected by FDR. Paralogs are binned into equal-sized bins. Left-most bins represent the highest dS values, and likely the oldest duplicates, while right-most bins represent lowest dS values. (***P < 0.001, **P < 0.01, *P < 0.05). Group size numbers appear above each box.

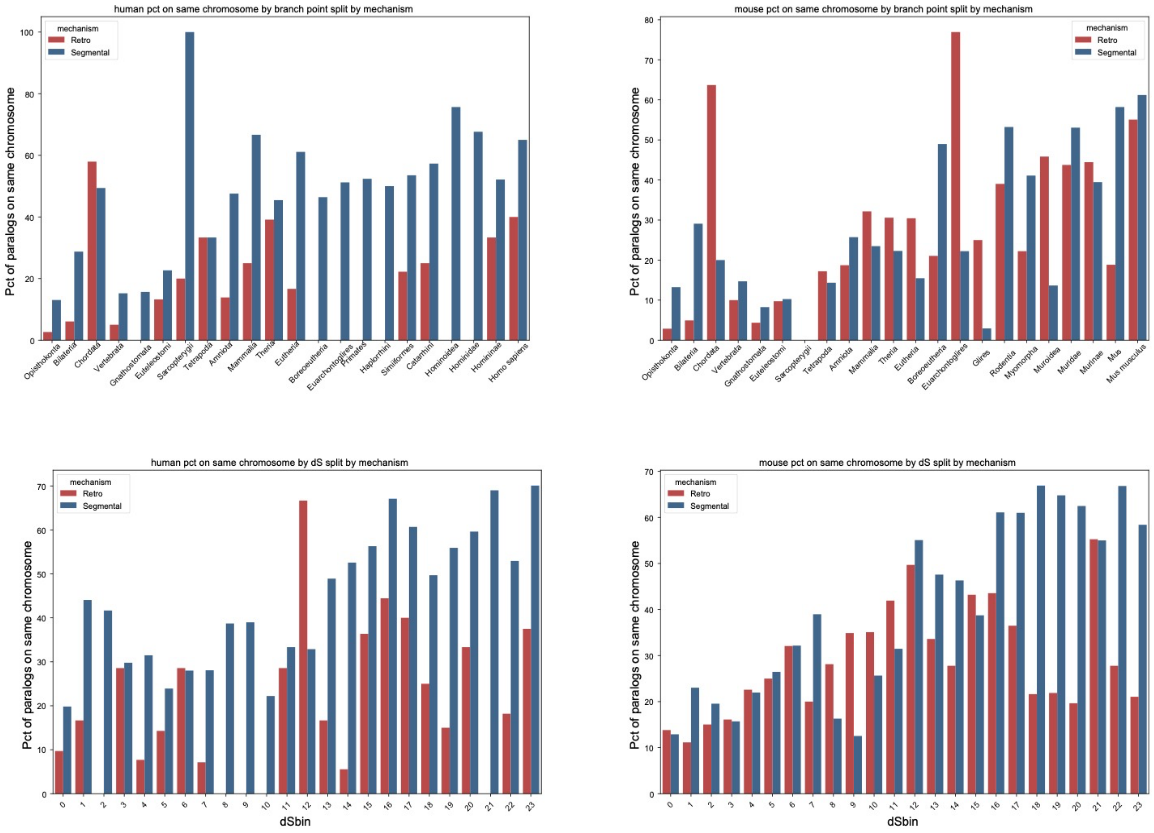

Supporting Figure 8: Percentage of gene duplicates that reside in the same chromosome. Top: Human duplicates partitioned by duplication time and by duplication mechanism. Bottom: Mouse duplicates partitioned by duplication time and by duplication mechanism (as in Figure 2).Red: retrotransposition-mediated, Blue: Segmental duplications. Group sizes are as in Fig 2A-B.

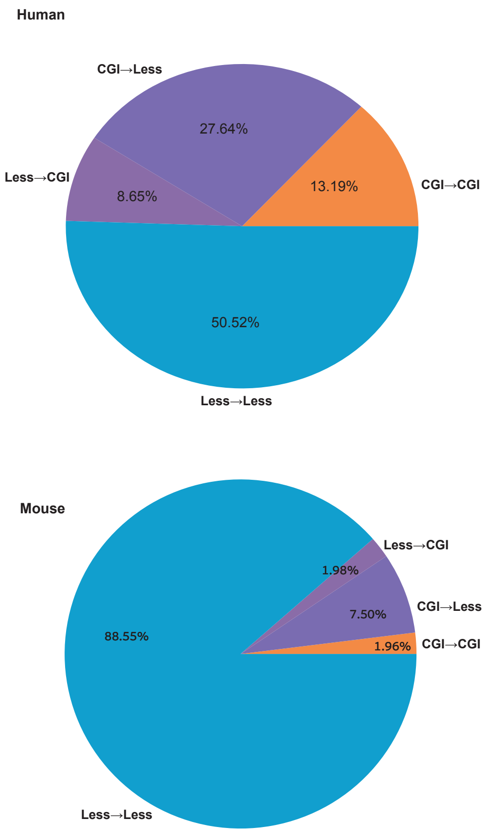

Supporting Figure 9: Partition of retrotransposition-mediated duplications by promoter architecture.

Pie charts showing the partition of gene duplicates through retrotransposition in human and mouse (that duplicated in both ancient and recent evolutionary periods), based on the promoter architecture of the original and the retro-copied genes. Each gene in these pairs can either be a CGI gene or a CGI-less gene, yielding four possible combinations. The combination is denoted using an arrow pointing from the original gene to the retro-copy gene (for example, CGI->CGI-less denotes the fraction of gene pairs that have CGI in promoters of the original gene and are depleted of CGI in promoters of the retro-copied gene). Group sizes are as in Fig 2A-B.

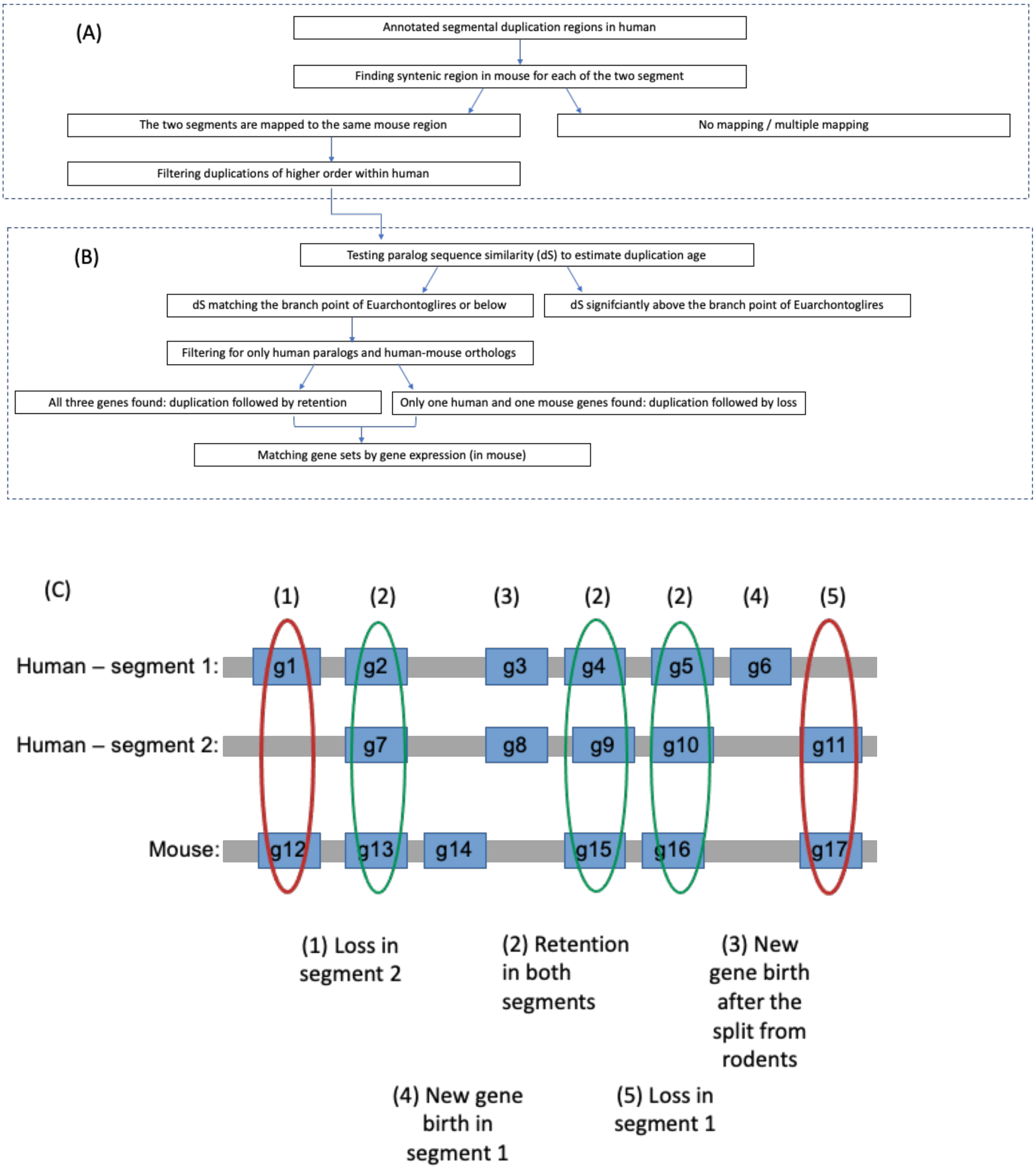

Supporting Figure 10: Work flow to obtain comparable sets of gene duplicates that were retained or lost in human segmental duplication regions. (A) Obtaining a clean set of segmental duplication regions in human and their syntenic regions in mouse: Starting from annotated segmental duplication regions from the UCSC Genome Browser, we searched for syntenic regions in mouse (using liftOver). All pairs that did not map uniquely to a single mouse region were filtered. Additionally, we filtered triple and higher-order segmental duplication. **(B)** Obtaining a clean set of segmental duplication events that resulted in gene retention or loss. Following the stages described in A, we scanned all genes that are annotated in the filtered set of segmental duplication regions in human and the syntenic region in mouse. In cases where we found two human paralogs – one in each segment, we tested

whether they are likely to have duplicated in the expected evolutionary time based on their dS (synonymous substitution rate). We then also filtered any human or mouse genes that were not annotated with the expected evolutionary relationship (i.e., orthologs between human and mouse genes and paralogs between the two duplicates in human). In all cases we required the human genes to have an ortholog in mouse. We were then left with two scenarios: a pair of duplicate gene in humans that represents a duplication followed by retention, and a singleton gene in one of the human segments that represents a duplication followed by loss. **(C)** Visualization of various scenarios of gene sets found in the two human segments and in the syntenic mouse segment. Genes found in a single column represent orthologs / paralogs.

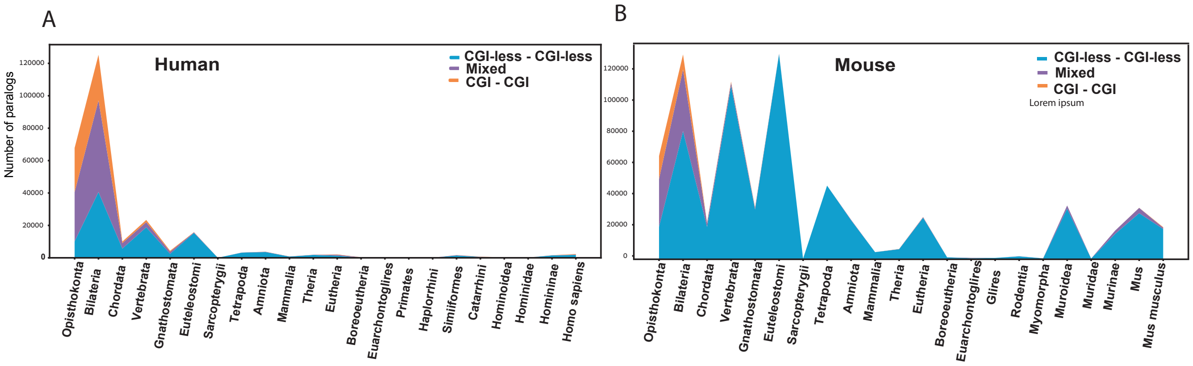

Supporting Figure 11: CGI and CGI-less genes duplication over evolutionary time.
(A) A timeline showing the numbers of human paralogs at each TMRCA from Opisthokonta to Homo Sapiens, where each point is split into CGI paralogs, CGI-less paralogs, and Mixed paralogs. **(B)** The same as in (A), only with mouse paralogs. CGI and Mixed pairs are skewed towards ancient times of duplication in both human and mouse paralogs (permutation test, $P<10^{-5}$). Group sizes are as in Fig 1A-B.

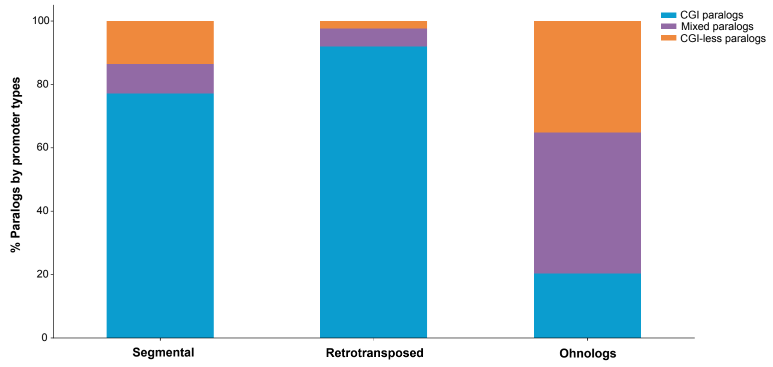

Supporting Figure 12: Distribution of CGI and CGI-less duplicates in different types of duplication mechanisms.

Human paralogs distribution, partitioned based on their promoter classification: CGI - CGI paralogs (orange), CGI-less – CGI-less paralogs (blue) and Mixed (purple) in paralogs resulting in different types duplication mechanisms. Left and central bars: recent paralogs, that are inferred to have duplicated after the split between primates and rodents, and that were identified to have duplicated through retrotranspositions or through other small-scale duplication mechanisms (segmental and tandem duplications) are shown in the central and left-most bars, respectively. Each bar includes the partition of these paralogs based on their promoter type. Duplication mediated by retrotranspositions have a lower fraction of CGI duplication in comparison with other recent duplications (P-value$<10^{-298}$, Chi-squared test). Right bar: Ohnologs (paralog products of WGD) split by their promoter type. Ohnologs are enriched with CGI duplications in comparison to all other types of duplications and in contrast to recent duplications that occurred through small-scale duplication mechanisms (P-value$<10^{-298}$, Chi-squared test).

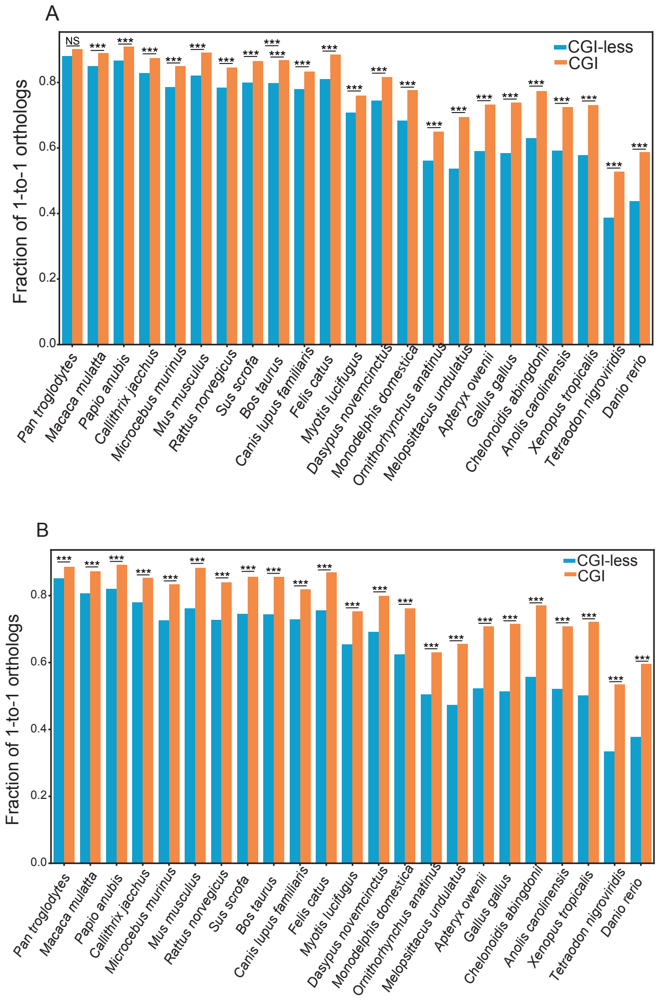

Supporting Figure 13: CGI and CGI-less gene duplication across species.

Fraction of 1-to-1 orthologs of human CGI and CGI-less genes with a selected number of species, ranging from chimpanzee to zebrafish. In each pair of compared species (human versus another vertebrate), the fractions of these one-to-one orthologs out of all human genes, which are either CGI and CGI-less genes, were compared (the size of group of CGI genes having orthologs, out of the group of all CGI genes, versus the size of group of CGI-less genes having orthologs, out of all CGI-less genes) using a chi-square test. P-values were corrected by FDR (*** - P<0.001). In (**A**) CGI and CGI-less genes are matched in each group by expression levels, in (**B**) genes are matched by gene length, to avoid these potential confounders. Group sizes are as in Fig 1A-B.

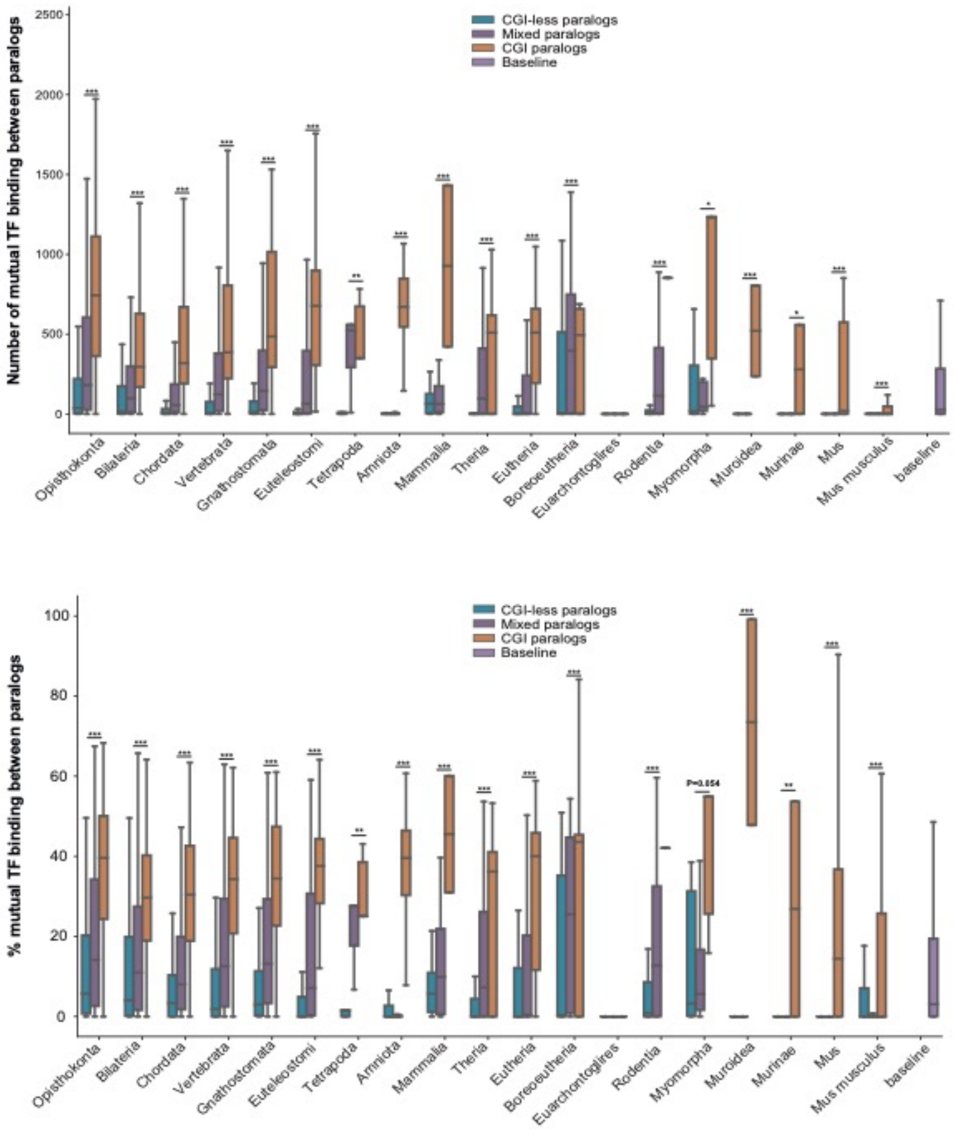

Supporting Figure 14: TF-binding in promoters of CGI and CGI-less genes and paralogs.
(A) A timeline showing the total number of TFs that bind to promoters of both paralogs. Mouse paralogs are split based on their inferred time to most common recent ancestor (TMRCA) - from Opisthokonta to Mus musculus, and are further split based on their promoter classification: CGI - CGI paralogs (orange), CGI-less – CGI-less paralogs (blue) and Mixed paralogs (purple), as well as baseline. In each inferred time, CGI and CGI-less paralogs are paired by expression, to control for gene expression level. **(B)** As in (A), only in fractions – the percentage of TFs that bind to promoters of both paralogs, out of the total number of TFs that bind to either of the promoters of the two paralogs. Comparison between the distributions of CGI paralogs and CGI-less paralogs was preformed using a one-sided Mann-Whitney test, and corrected by FDR. (***P < 0.001, **P < 0.01, *P < 0.05). Group sizes are as in Fig 1A-B.

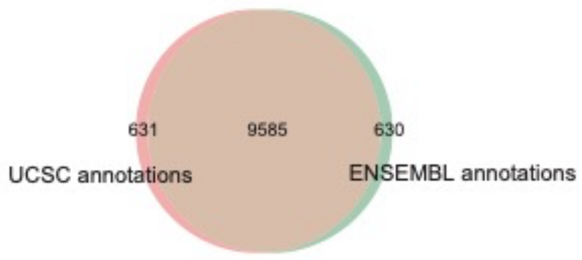

Supporting Figure 15: Agreement of CGI annotations between UCSC and ENSEMBL. Different sources of annotations of CGIs yield similar CGI gene sets: Annotated CGI regions from ENSEMBL and from UCSC were overlapped with promoter regions of genes. Genes were defined as CGI genes if their promoter region overlapped with an annotated CGI for at least 50% of its length. The results show that the CGI annotations from UCSC and ENSEMBL give similar CGI gene sets (88.4% of the genes are shared).

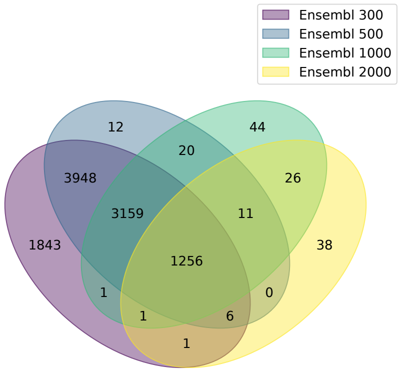

Supporting Figure 16: Agreement of CGI gene definitions when using different promoter length definitions to overlap with CGI regions. Annotated CGI regions from ENSEMBL were overlapped with promoter regions of genes. Genes were defined as CGI genes if their promoter region overlapped with an annotated CGI for at least 50% of its length. The promoter region varied between 300 to 2,000bp upstream and 100bp downstream of the TSS, showing a large agreement between the CGI gene definitions across different promoter lengths.
